# Supplementary material for: Identification of sporulation genes in Bacillus anthracis highlights similarities and significant differences with Bacillus subtilis
Source: PLoS Biol. 2025 Dec 12;23(12):e3003521. doi: 10.1371/journal.pbio.3003521 (PMC12700454; doi:10.1371/journal.pbio.3003521)
Supplement: S1 Raw Images — (PDF) [file pbio.3003521.s010.pdf]

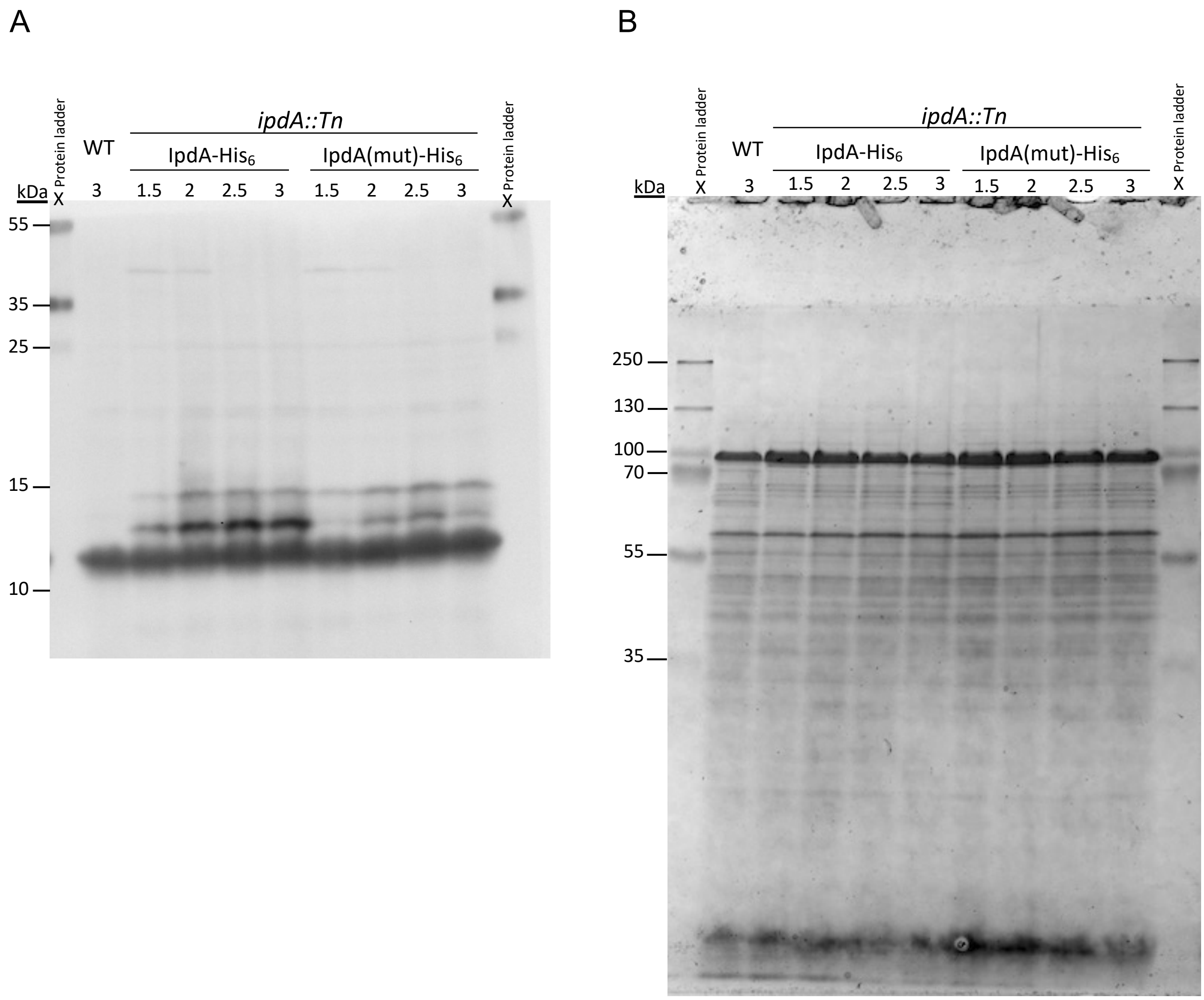

S1 Raw Images. These are the raw images that correspond to the images shown in **Fig 5J. A)** Anti-His blot. Signal was detected using a ChemiDocTM MP, Imagin System (BIO-RAD) with an exposure time of 60 s. **B)** Coomassie Brillant Blue stained gel. Image was acquired with the same equipment with autoexposure. Protein ladder (PageRuler Plus) is indicated in the blot and the gel. X indicates lanes that were not included in the final figure.
