# Supplementary material for: Identification of sporulation genes in Bacillus anthracis highlights similarities and significant differences with Bacillus subtilis
Source: PLoS Biol. 2025 Dec 12;23(12):e3003521. doi: 10.1371/journal.pbio.3003521 (PMC12700454; doi:10.1371/journal.pbio.3003521)
Supplement: S1 Text — Plasmid constructions. Table A. List of Bacillus strains used in this study. Table B. List of plasmids used in this study. Table C. List of oligonucleotide primers used in this study. Fig A. Examples of genes required for B. anthracis sporulation that are also critical for B. subtilis sporulation. Transposon-insertion profiles from seven regions of the B. anthracis genome. Each contains a gene (or genes) that encode B. subtilis homologs that are required for B. subtilis sporulation. Each vertical line represents a transposon-insertion site, and its height reflects the relative number of insertions mapped to that site. The maximum height for each panel was set to the indicated number of reads shown in the right corner. Transposon-insertions in B. anthracis sigF, spoIIAB (IIAB), spoIIAA (IIAA), spoIIID (IIID), spoIIQ, spoIIIE, spoIVA, spoIVFB, spoIVFA, spoVD, and spoVV were underrepresented after sporulation, heat treatment, germination, and outgrowth compared to the input library. Fig Ba. Examples of transposon-insertion profiles of B. anthracis and B. subtilis genes that are critical for sporulation in B. anthracis but not in B. subtilis. Transposon-insertion profiles from three regions of the B. anthracis genome and the corresponding regions of the B. subtilis genome. Each line represents a transposon-insertion site, and its height reflects the relative number of insertions mapped to that site. The maximum height for each panel was set to 200 reads. Transposon-insertions in yyaC, yqfT (T), and yhfN were underrepresented after sporulation, heat treatment, germination, and outgrowth in B. anthracis compared to the input library (onset). By contrast, transposon-insertions in the homologous genes (dark gray) in B. subtilis were not underrepresented after sporulation, heat treatment, germination, and outgrowth. The B. subtilis Tn-seq data are from Meeske and colleagues 2016 [17]. Fig Bb. Examples of transposon-insertion profiles of B. anthracis and B. subtilis genes tha [file pbio.3003521.s011.pdf]

SUPPLEMENTARY INFORMATION

Identification of sporulation genes in *Bacillus anthracis* highlights similarities and significant differences with *Bacillus subtilis*

Fernando H. Ramírez-Guadiana, Anna P. Brogan, Yuanchen Yu, Caroline Midonet, Joel W. Sher, Ernst W. Schmid, Ian J. Roney, and David Z. Rudner

Department of Microbiology, Harvard Medical School
77 Avenue Louis Pasteur. Boston, Massachusetts

Supporting information includes:

Supplemental Methods
Figures A to T
Tables A to C

SUPPLEMENTAL METHODS

Plasmid constructions

pFR38 [Himar1C9 IR-Spec (*amp, erm, spec*)] was constructed via two-way ligation of a PstI-HindIII fragment —containing a spectinomycin resistance cassette flanked by two inverted terminal repeats, one of which contained an MmeI site, into pMarA digested with the same enzymes.

pFR50 [pMiniMAD_P_{veg}-*mCherry* (*amp*, *erm*)] was constructed via two-way ligation of an EcoRI-BamHI fragment containing the *veg* promoter from *B. subtilis* fused to *mCherry* into pER75 digested with the same enzymes. pER75 [*sacA*::P_{veg}-*mCherry* (kan)] is a double crossover vector at the *sacA* locus (Rudner Lab stock).

pFR76 [*yvbJ*::P_{spank}-*optRBS-pdaN (kan)*] was constructed in a two-piece isothermal assembly reaction using a PCR product containing *pdaN* amplified from *B. anthracis* BaR1 with oligonucleotide primers oFR618 and oFR619 and pCB121 cut with SpeI and PstI. pDR121 [*yvbJ*::P_{spank}-kan] is a double crossover integration vector with an IPTG-inducible promoter for ectopic integration at the *yvbJ* locus (Rudner Lab stock).

pFR77 [*amyE*::P_{spank}-*optRBS-pdcF (spec)*] was constructed in a two-way ligation with a SalI-SphI fragment containing the *pcdF* gene from *B. anthracis* BaR1 and pDR110 digested with the same enzymes. The insert contains an optimized amino acid sequence of *B. anthracis pdcF* to improve expression levels in *B. subtilis.* pDR110 [*amyE*::P_{spank}-spec] is a double crossover vector with an IPTG-inducible promoter for ectopic integration at the *amyE* locus (Rudner Lab stock).

pBaR5 [pMiniMAD_P_{veg}-*mCherry* 395/396::*kan* (*amp, erm*] was assembled in two steps. First, two fragments of 1,019 bp spanning the intergenic region between the convergently transcribed genes *00395* and *00396* were PCR-amplified from *B. anthracis* BaR1 with oligonucleotide primer pairs oFR295/oFR296 and oFR297/oFR298. These PCR products correspond to upstream (BamHI-ApaI) and downstream (ApaI-SalI) flanking regions, respectively. The two fragments were ligated into pFR50 digested with BamHI and SalI. In the second step, an kanamycin resistance cassette, flanked by strong transcriptional terminators, was cut with ApaI and inserted into the intermediate construct digested with ApaI.

pBaR8 [pMiniMAD_P_{veg}-*mCherry* 380/381::*cat* (*amp, erm*)] was assembled in two steps. First, two fragments spanning the intergenic region between the convergently transcribed genes *00380* and *00381* were PCR-amplified from *B. anthracis* BaR1 using oligonucleotide primer pairs oFR299/oFR300 and oFR301/oFR302. These fragments correspond to the 1,027 bp upstream (BamHI-ApaI) and 1,018 bp downstream (ApaI-SalI) flanking regions, respectively. Both fragments were ligated into pFR50 digested with BamHI and SalI. In the second step, a chloramphenicol resistance cassette, flanked by strong transcriptional terminators, was cut with ApaI and inserted into the intermediate construct digested with ApaI.

pBaR9 [pMiniMAD_P_{veg}-*mCherry* 395/396::P_{spoIID}-*optRBS-cfp* (*kan*) (*amp, erm*)] was assembled via a three-way ligation with (1) a KpnI-EcoRI PCR product containing the SigE-responsive *spoIID* promoter (amplified with oligonucleotide primers oFR309 and oFR310 using *B. anthracis* BaR1 gDNA as template), (2) an EcoRI-NheI PCR product containing an optimized ribosome binding site (*optRBS*) fused to a *cfp* reporter (amplified with oligonucleotide primers oFR311 and oFR312 using DNA from pCB137 as template). These two fragments were ligated into pBaR5 digested with KpnI and NheI. pCB137 [*yycR*::P_{sspB}-*optRBS-cfp* (*spec*)] is a double-crossover integration vector for ectopic integration

at the *yycR* locus. It carries the *sspB* promoter from *B. subtilis* fused to an *optRBS-cfp* reporter (Rudner Lab stock).

pBaR10 [pMiniMAD_P_{veg}-*mCherry* 380/381::P_{spoIIQ}-*optRBS-yfp* (*cat*) (*amp, erm*)] was assembled via a three-way ligation of: (1) a KpnI-EcoRI PCR product containing the SigF-responsive *spoIIQ* promoter (amplified with oligonucleotide primers oFR314 and oFR315 using *B. anthracis* BaR1 gDNA as template), and (2) an EcoRI-SpeI PCR product containing an *optRBS* fused to a *yfp* reporter (amplified with oligonucleotide primers oFR311 and oFR320 using DNA from pCB138 as template). Both fragments were ligated into pBaR8 digested with KpnI and SpeI. pCB138 [*yycR*::P_{sspB}-*optRBS-yfp* (*spec*)] is a double-crossover vector for ectopic integration at the *yycR* locus, carrying the *sspB* promoter from *B. subtilis* fused to an *optRBS-yfp* reporter (Rudner Lab stock).

pBaR20 [pMiniMAD_P_{veg}-*mCherry* 395/396::P_{ipdA}-*optRBS-yfp* (*kan*) (*amp, erm*)] was assembled via a three-way ligation of: (1) a KpnI-EcoRI PCR fragment containing the *ipdA* promoter (amplified with oligonucleotide primers oFR465 and oFR466 using *B. anthracis* BaR1 gDNA as template), and (2) an EcoRI-SpeI PCR fragment containing an *optRBS* fused to a *yfp* reporter (amplified with oligonucleotide primers oFR311 and oFR320 using DNA from pCB138 as template). Both fragments were ligated into pBaR5 digested with KpnI and SpeI.

pBaR28 [pMiniMAD_P_{veg}-mCherry Δ*ipdA::kan* (*amp, erm*)] was assembled in two steps: first, two fragments of 1,009 and 985 bp spanning the ends of the *ipdA* gene were PCR-amplified from *B. anthracis* BaR1 with oligonucleotide primer pairs oFR527/oFR528 and oFR531/oFR532, respectively. These fragments corresponded to the upstream region (SalI–ApaI), including the first five codons of *ipdA*, and the downstream region (ApaI–BamHI), including the last five codons of *ipdA*. In the second step, an ApaI-ApaI kanamycin resistance cassette, flanked by strong transcriptional terminators, was inserted into the intermediate construct digested with ApaI.

pBaR36 [pMiniMAD_P_{veg}-*mCherry* 395/396::*ipdA* (*kan*) (*amp, erm*)] was constructed via two-way ligation of a KpnI-NheI fragment —containing the *ipdA* gene PCR-amplified from *B. anthracis* BaR1 with oligonucleotide primers oFR554 and oFR555—, into pBaR5 digested with the same enzymes.

pBaR39 [pMiniMAD_P_{veg}-*mCherry* 395/396::P_{pdaN}-*optRBS-yfp* (*kan*) (*amp, erm*)] was assembled via a three-way ligation of: (1) a KpnI-EcoRI PCR fragment containing the *pdaN* promoter (amplified with oligonucleotide primers oFR564 and oFR565 using *B. anthracis* BaR1 gDNA as template), and (2) an EcoRI-SpeI PCR fragment containing an *optRBS* fused to a *yfp* reporter (amplified with oligonucleotide primers oFR311 and oFR320 using DNA from pCB138 as template). Both fragments were ligated into pBaR5 digested with KpnI and SpeI.

pBaR40 [pMiniMAD_P_{veg}-*mCherry* 395/396::P_{pdcF}-*optRBS-yfp* (*kan*) (*amp, erm*)] was assembled via a three-way ligation of: (1) a KpnI-HindIII PCR fragment containing the *pdcF* promoter (amplified with oligonucleotide primers oFR566 and oFR567 using *B. anthracis* BaR1 gDNA as template), and (2) an HindIII-SpeI PCR fragment containing an *optRBS* fused to a *yfp* reporter (amplified with oligonucleotide primers oFR320 and oFR568 using DNA from pCB138 as template). Both fragments were ligated into pBaR5 digested with KpnI and SpeI.

pBaR44 [pMiniMAD_P_{veg}-*mCherry* 395/396::*ipdA-his*$_6$ (*kan*) (*amp, erm*)] was constructed via two-way ligation of a KpnI-NheI fragment —containing the *ipdA* gene with a C-terminal hexahistidine tag PCR-amplified from *B. anthracis* BaR1 with oligonucleotide primers oFR587 and oFR554—, into pBaR5 digested with the same enzymes.

pBaR47 [pMiniMAD_P$_{veg}$-*mCherry* 395/396::*ipdA*(N73A,Q76A,D80A)-*his*$_{6}$ (*kan*) (*amp, erm*)] was generated via site-directed using oligonucleotide primers oFR590 and oFR591, with pBaR44 as the template.

***pdcF* gBlock sequence:**
gcc**GTCGAC***acaTAAGGAGGaactact*ATGCTTAAGTATAGTAAACTCGCCATCGTCACTGCGTTATCC
ATGACGTTACTGGCAGGTTGTTTTGGGCCAAAACCAGAGGAAGAGTTGTACGTAGCCTTCGAAAA
CGCTGCGAAACAGGAGAAAACGATGTTTGAGGATGCTAAAAAGTTGGAGACTCTTGAGAAAGAA
GGCCAGGAACTCTACAATCAGATCGTACAAGAGGGCAAGGACAATAATCAAACCGTGAAAGAGAA
GCTTAACCAGGCCGTTAAGAATACTGACGAAAGAGAAAAAGTCCTGAAGAAAGAAAAGGAGAGCT
TAAATAAAGCTCAAGAGGAGGTGAAGAGCGCCGATAAGTACGTCAAGAAGATTGAGGATAAAAAG
TTGAAAGATCAAGCCGACAAAGTCAAATCCACGTATGAGAAACGTCATGACAGCTTCAATAAAATG
TATGACTCCTACAACAAGTCCCTGAAGCAAGAAAAGGAGCTTTATACAATGTTGCAAGATAAGGGA
ACTAAGCTGAAGGATATCTCAGAGAAGGTGAAGGTAGTAAATCAATCATATAAAGACATTGACTCA
GAGAAGGACAAATTCAACGAATTCACAAAGAGTTACAACACAGAGAAGATAGCATTTTATAAGCAG
GCAAACATCAAGATAAAGGAAGAGAAAAAGTAAaacagtttggcctacaattggccaaact**GCATGC**cgg

SalI and PstI restriction sites are highlighted in bold. The optRBS and the spacer are shown in italics.

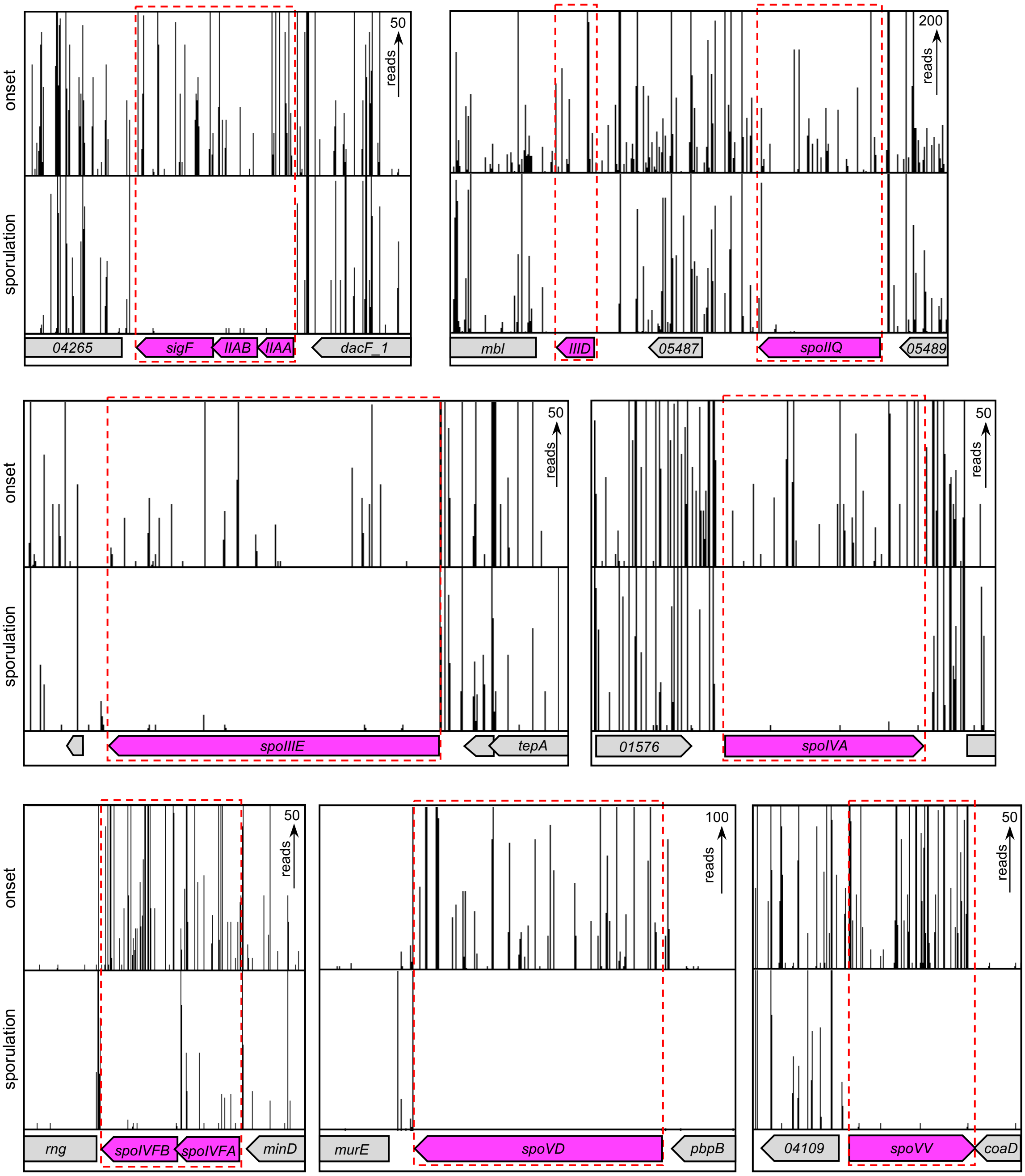

Figure A. Examples of genes required for *B. anthracis* sporulation that are also critical for *B. subtilis* sporulation. Transposon-insertion profiles from seven regions of the *B. anthracis* genome. Each contains a gene (or genes) that encode *B. subtilis* homologs that are required for *B. subtilis* sporulation. Each vertical line represents a transposon insertion site, and its height reflects the relative number of insertions mapped to that site. The maximum height for each panel was set to the indicated number of reads shown in the right corner. Transposon insertions in *B. anthracis sigF*, *spoIIAB* (*IIAB*), *spoIIAA* (*IIAA*), *spoIIID* (*IIID*), *spoIIQ*, *spoIIIE*, *spoIVA*, *spoIVFB*, *spoIVFA*, *spoVD* and *spoVV* were underrepresented after sporulation, heat treatment, germination, and outgrowth compared to the input library.

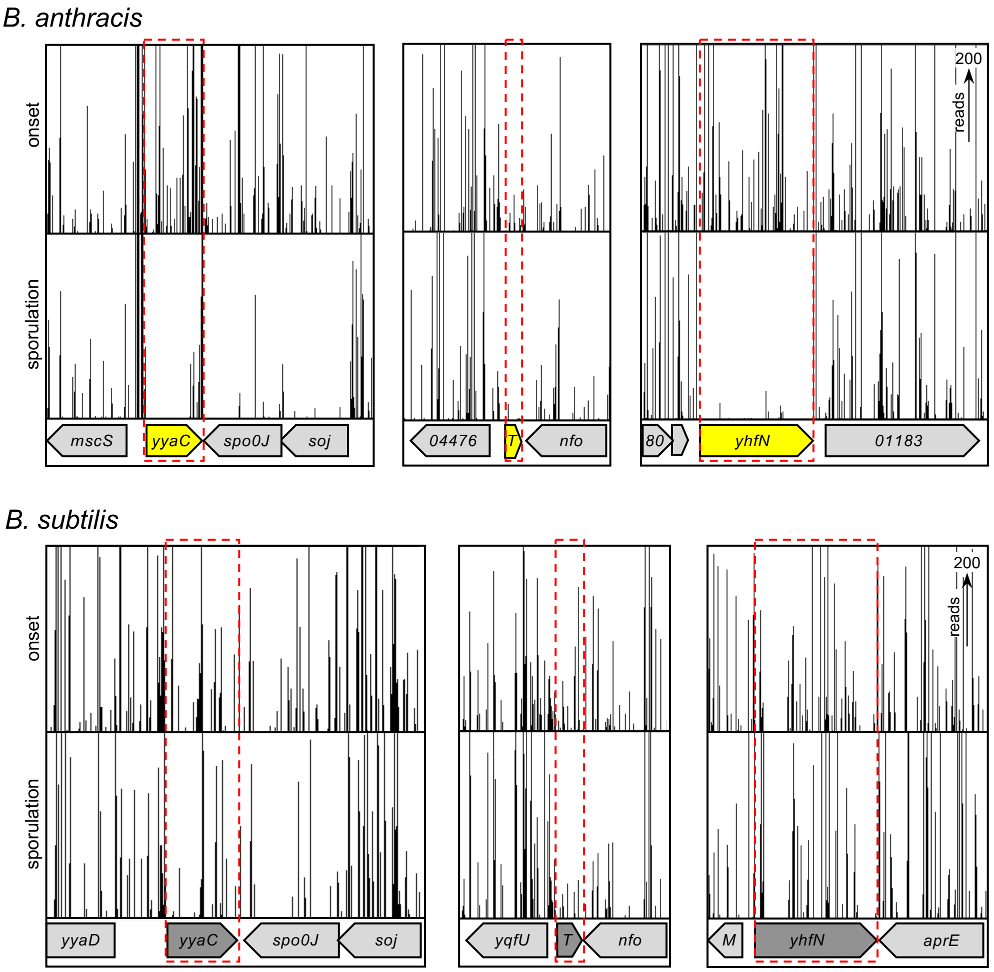

Figure Ba. Examples of transposon insertion profiles of *B. anthracis* and *B. subtilis* genes that are critical for sporulation in *B. anthracis* but not in *B. subtilis*. Transposon-insertion profiles from three regions of the *B. anthracis* genome and the corresponding regions of the *B. subtilis* genome. Each line represents a transposon insertion site, and its height reflects the relative number of insertions mapped to that site. The maximum height for each panel was set to 200 reads. Transposon insertions in *yyaC*, *yqfT* (*T*) and *yhfN* were underrepresented after sporulation, heat treatment, germination, and outgrowth in *B. anthracis* compared to the input library (onset). By contrast, transposon insertions in the homologous genes (dark grey) in *B. subtilis* were not underrepresented after sporulation, heat treatment, germination, and outgrowth. The *B. subtilis* Tn-seq data are from *Meeske et al 2016.*

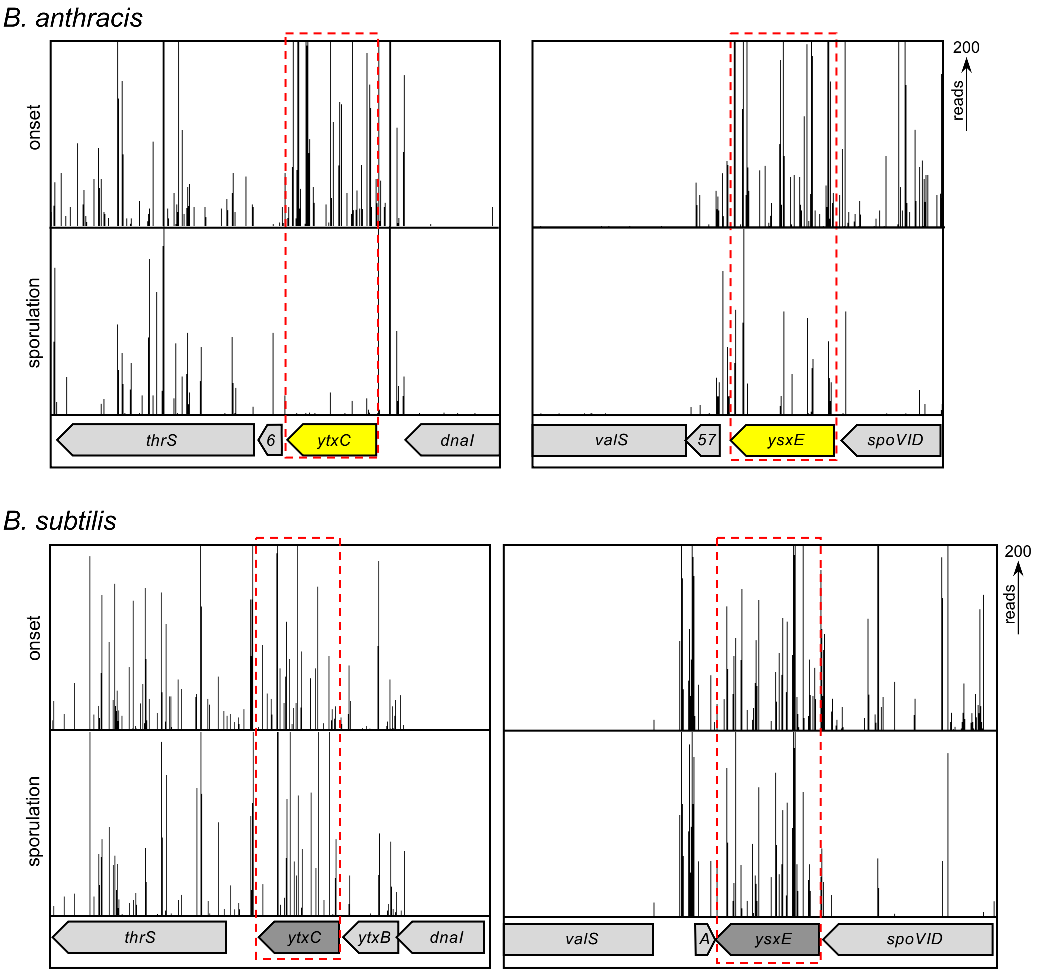

Figure Bb. Examples of transposon insertion profiles of *B. anthracis* and *B. subtilis* genes that are critical for sporulation in *B. anthracis* but not in *B. subtilis*. Transposon-insertion profiles from two regions of the *B. anthracis* genome and the corresponding regions of the *B. subtilis* genome. Each line represents a transposon insertion site, and its height reflects the relative number of insertions mapped to that site. The maximum height for each panel was set to 200 reads. Transposon insertions in *ytxC* and *ysxE* were underrepresented after sporulation, heat treatment, germination, and outgrowth in *B. anthracis* compared to the input library (onset). By contrast, transposon insertions in the homologous genes (dark grey) in *B. subtilis* were not underrepresented after sporulation, heat treatment, germination, and outgrowth. The *B. subtilis* Tn-seq data are from *Meeske et al 2016.*

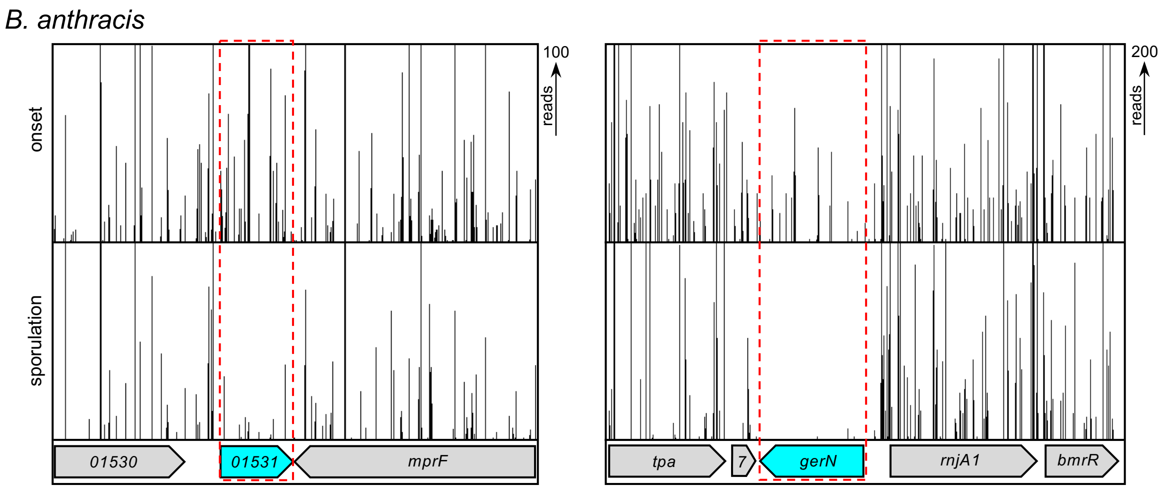

Figure C. Examples of *B. anthracis* sporulation genes that are not present in *B. subtilis*. Transposon-insertion profiles from two regions of the *B. anthracis* genome. Each line represents a transposon insertion site, and its height reflects the relative number of insertions mapped to that site. The maximum height for each panel was set to the indicated number of reads shown in the right corner. Transposon insertions in *01531* and *gerN* were underrepresented after sporulation, heat treatment, germination, and outgrowth in *B. anthracis* compared to the onset of starvation.

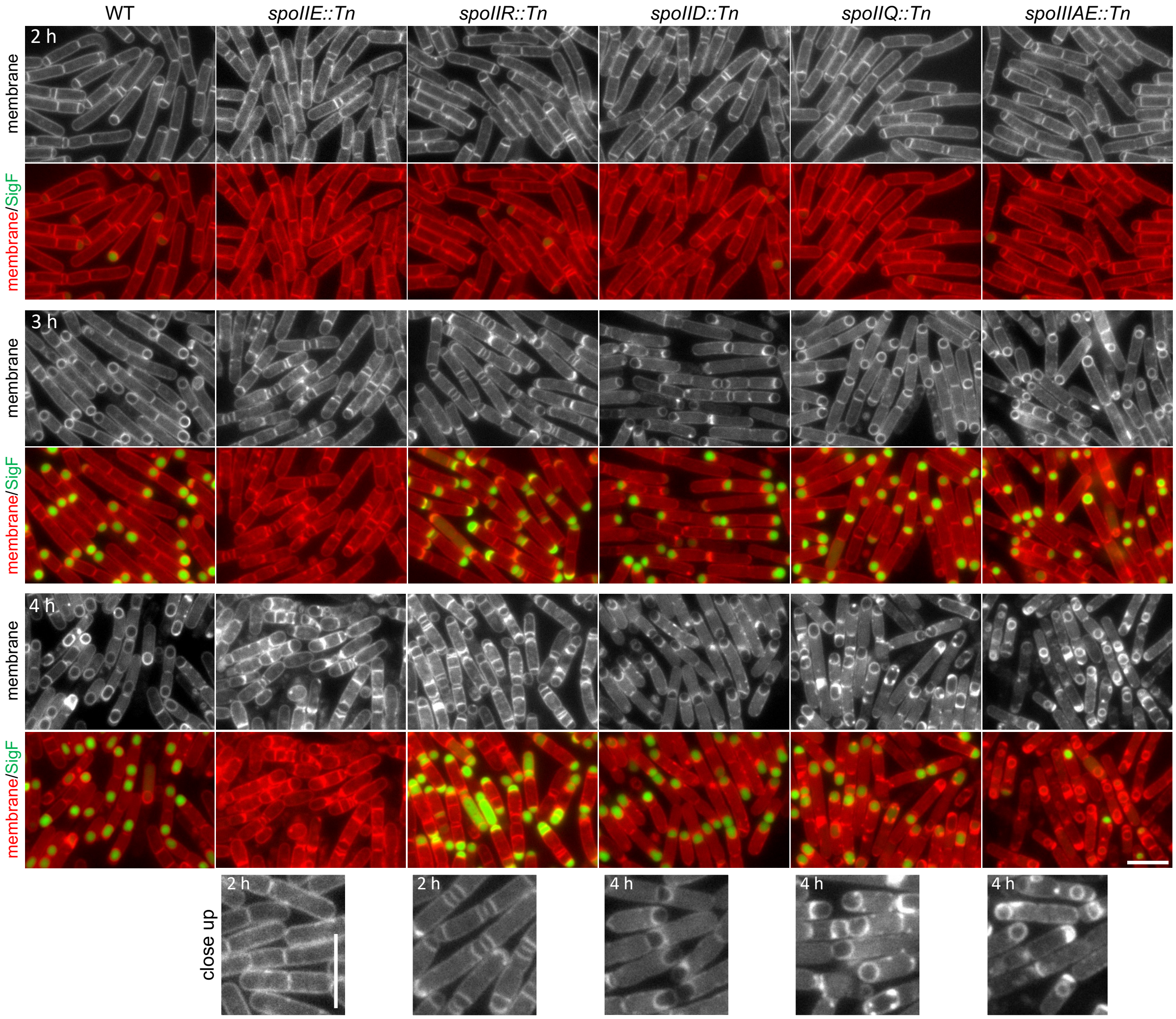

Figure D. Conserved *B. anthracis* sporulation genes exhibit mutant phenotypes that are similar, but not identical, to *B. subtilis* mutants. Representative fluorescence images of the indicated *B. anthracis* mutants during a sporulation time course. Time after the onset of sporulation is indicated in the top left corner. All strains harbor a forespore (P_{spoIIQ}-*yfp*) transcriptional reporter. σ^F (SigF) activity in the forespore was visualized with YFP (false-colored green). The membranes were stained with TMA-DPH (false-colored red in the merged images). The *B. anthracis spoIIE::Tn* mutant is delayed in polar division, fails to activate σ^F, and over time has impaired morphogenesis, similar to what has been reported for *B. subtilis* Δ*spoIIE.* The *B. anthracis spoIIR::Tn* mutant divides at both poles generating disporic sporangia. In some cases, both forespores activate σ^F, in others only one of the two forespores have σ^F activity. Over time, additional polar septa form. The *spoIID*::*Tn* mutant is impaired in engulfment and, in most cases, fails to complete it. However, unlike the *B. subtilis* Δ*spoIID* mutant, the *B. anthracis* mutant does not form characteristic septal membrane bulges. The *spoIIQ*::*Tn* mutant engulfs almost as well as wild-type, but a subset of cells fail to complete the process. The mutant displays heterogeneous morphologies with unusual membrane blebs. The *spoIIIAE::Tn* mutant produces small forespores. This phenotype is reminiscent of the collapsed forespores observed in *B. subtilis* Δ*spoIIIA* and Δ*spoIIQ* mutants. Finally, the YFP fluorescence from the σ^F reporter accumulates to wild-type levels at hour 3 of sporulation but is almost completely lost by hour 4. The reduction in expression of a σ^F-responsive gene is consistent with previous studies in *B. subtilis* that report a loss in metabolic potential at late timepoints in the absence of the *spoIIIA* locus. Scale bars indicate 5μm.

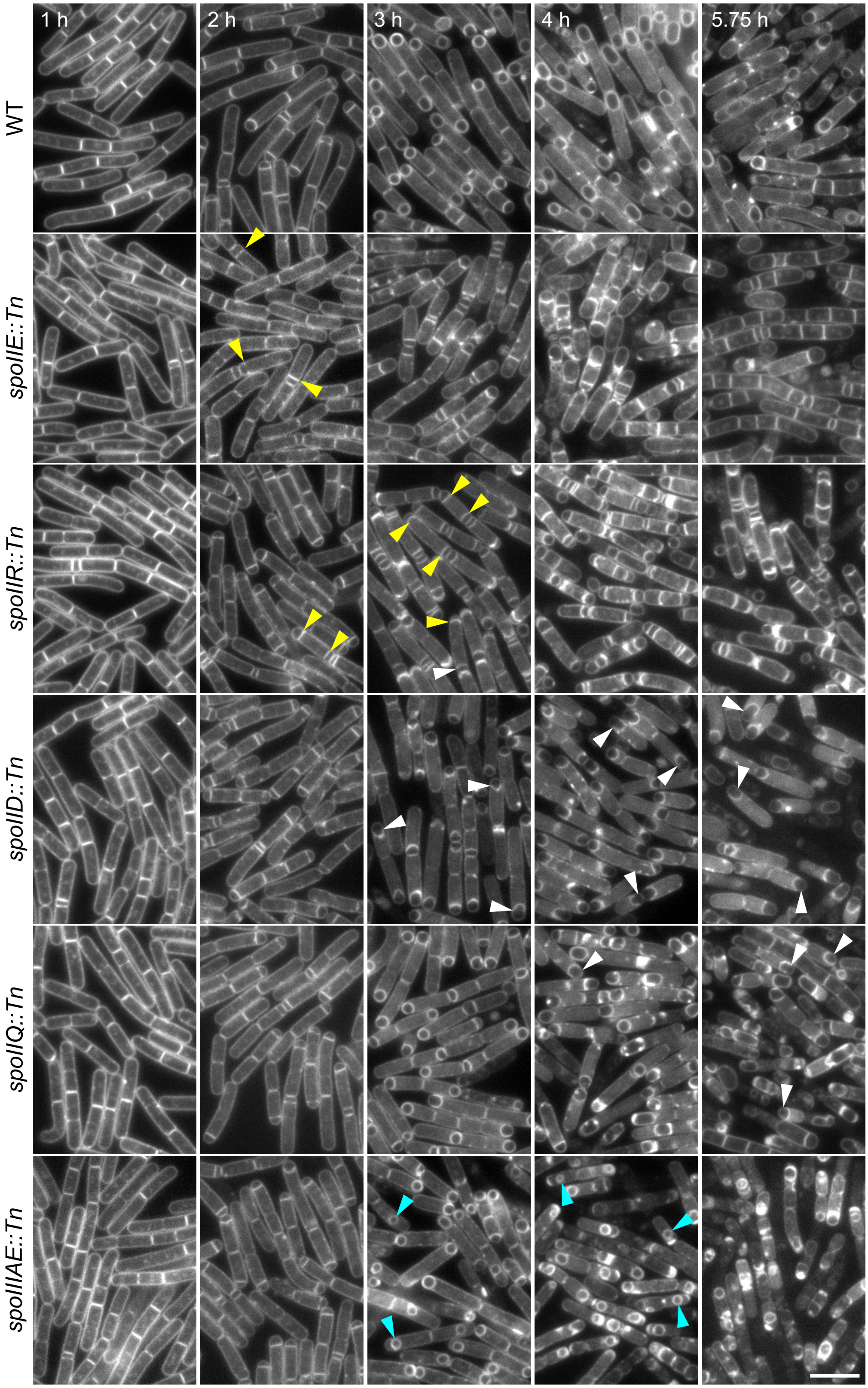

Figure E. Conserved sporulation genes have similar but not identical mutant phenotypes. Representative fluorescence images of the indicated *B. anthracis* sporulation mutants during a sporulation time course. Time after the onset of sporulation is indicated in the top left corner. Membranes were stained with TMA-DPH. Carets highlight unusual features. Scale bar indicates 5 μm.

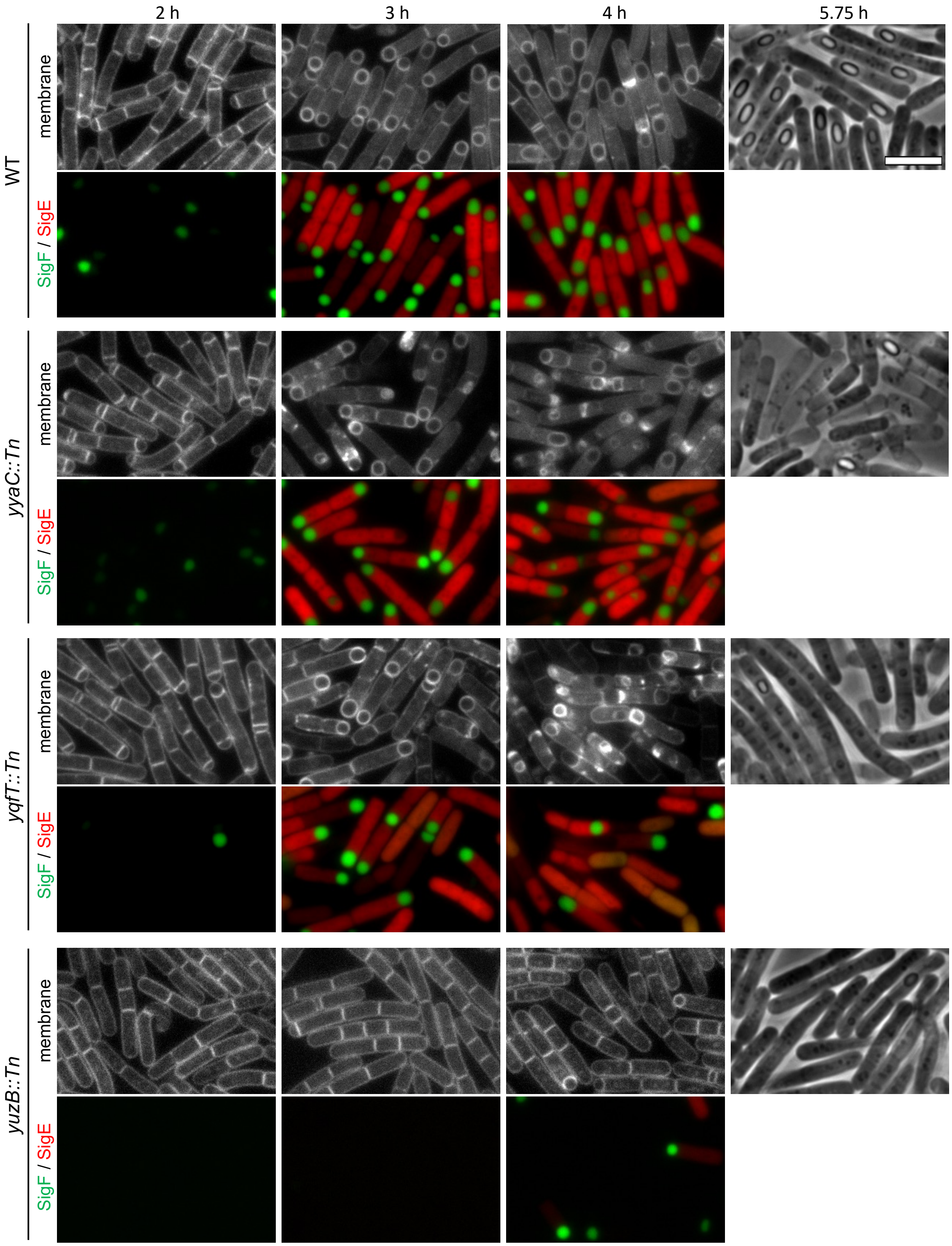

Figure Fa. Genes that are important for sporulation in *B. anthracis* but are not critical for spore formation in *B. subtilis*. Representative fluorescence images of the indicated *B. anthracis* mutants at the indicated times after the onset of sporulation. All strains have forespore and mother cell transcriptional reporters. The membranes were visualized with TMA-DPH. Scale bar indicates 5 μm.

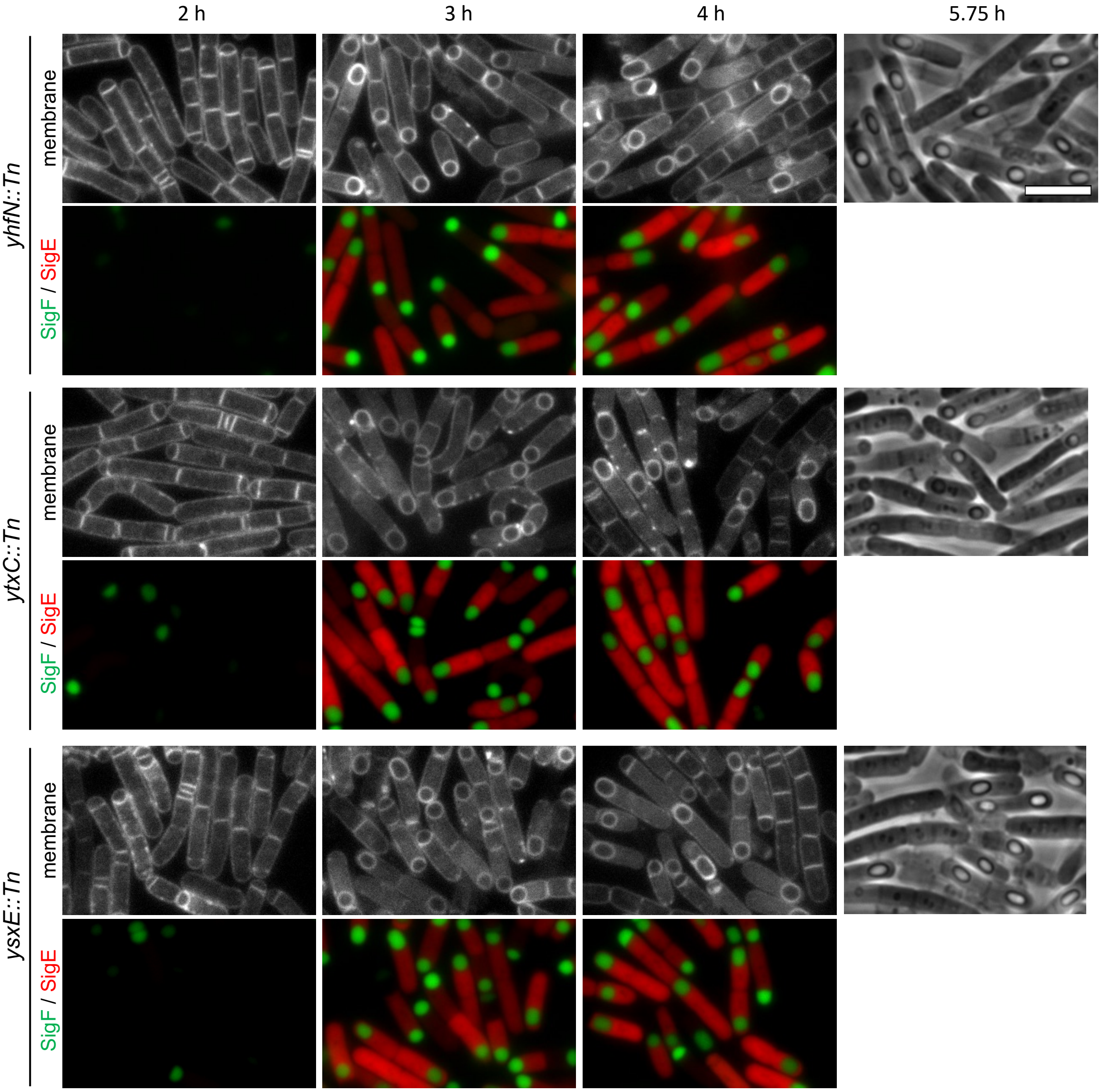

Figure Fb. Genes that are important for sporulation in *B. anthracis* but are not critical for spore formation in *B. subtilis*. Representative fluorescence images of the indicated *B. anthracis* mutants at the indicated times after the onset of sporulation. All strains have forespore and mother cell transcriptional reporters. The membranes were visualized with TMA-DPH. Scale bar indicates 5 μm.

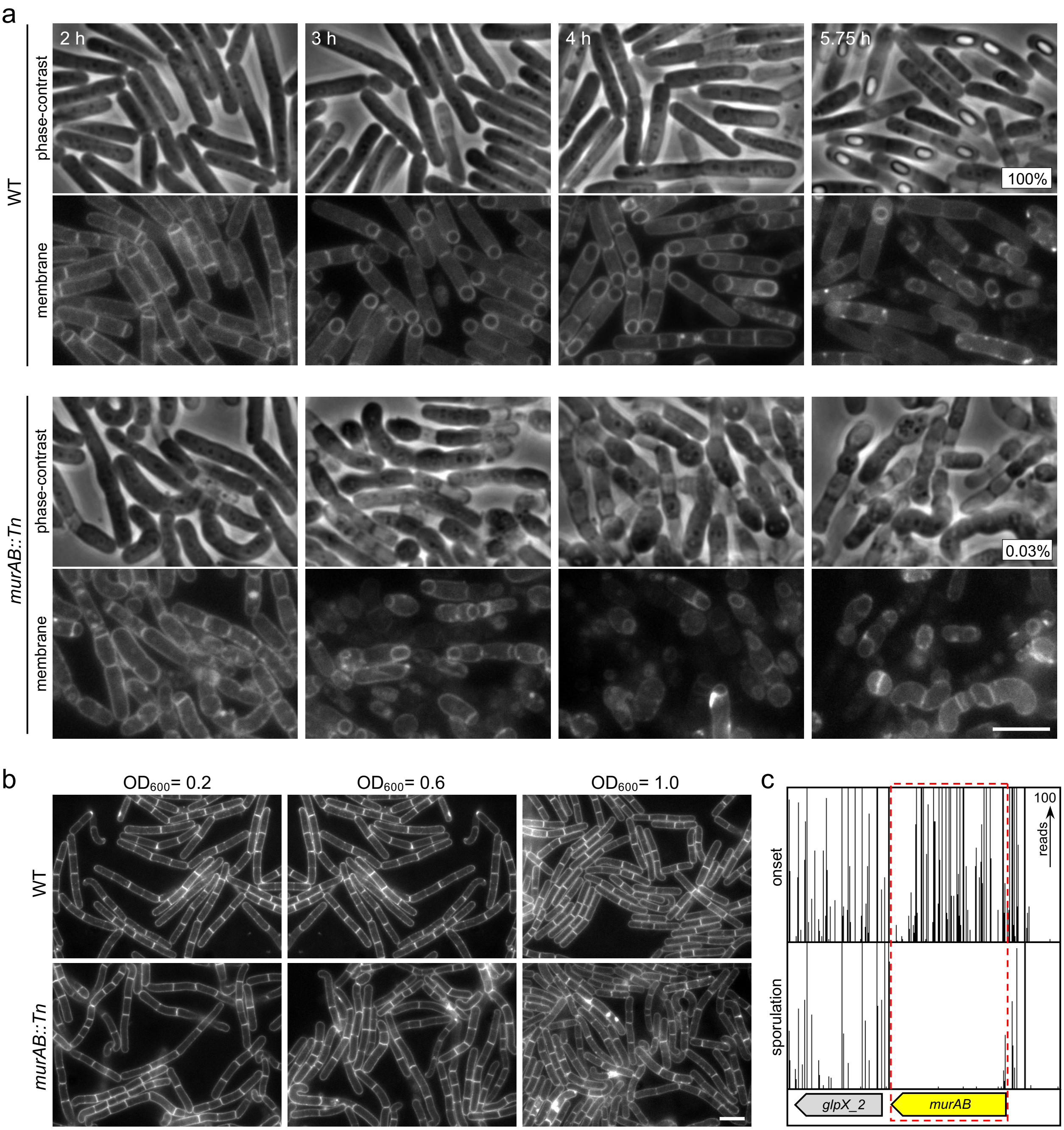

Figure G. MurAB is important for sporulation in *B. anthracis*. (a) Representative phase-contrast and fluorescence images of wild-type (WT) and *murAB::Tn* strains during a sporulation time course. Time after the initiation of sporulation in PA medium is indicated in the top left corner. The membranes were stained with TMA-DPH. The sporulation efficiencies of wild-type and the mutant determined in the BaR6 background are shown in the bottom right of the phase-contrast images. **(b)** Representative fluorescence images of WT and *murAB::Tn* during exponential growth in BHI medium. Optical densities are indicated above the images. Exponentially growing cells lacking *murAB* are slighter wider and curvy or bent compared to WT. Scale bars indicate 5 µm. **(c)** Transposon-insertion profile of the region containing the *murAB* gene. Insertions in *murAB* are underrepresented after sporulation, heat treatment, germination, and outgrowth compared to the onset of sporulation.

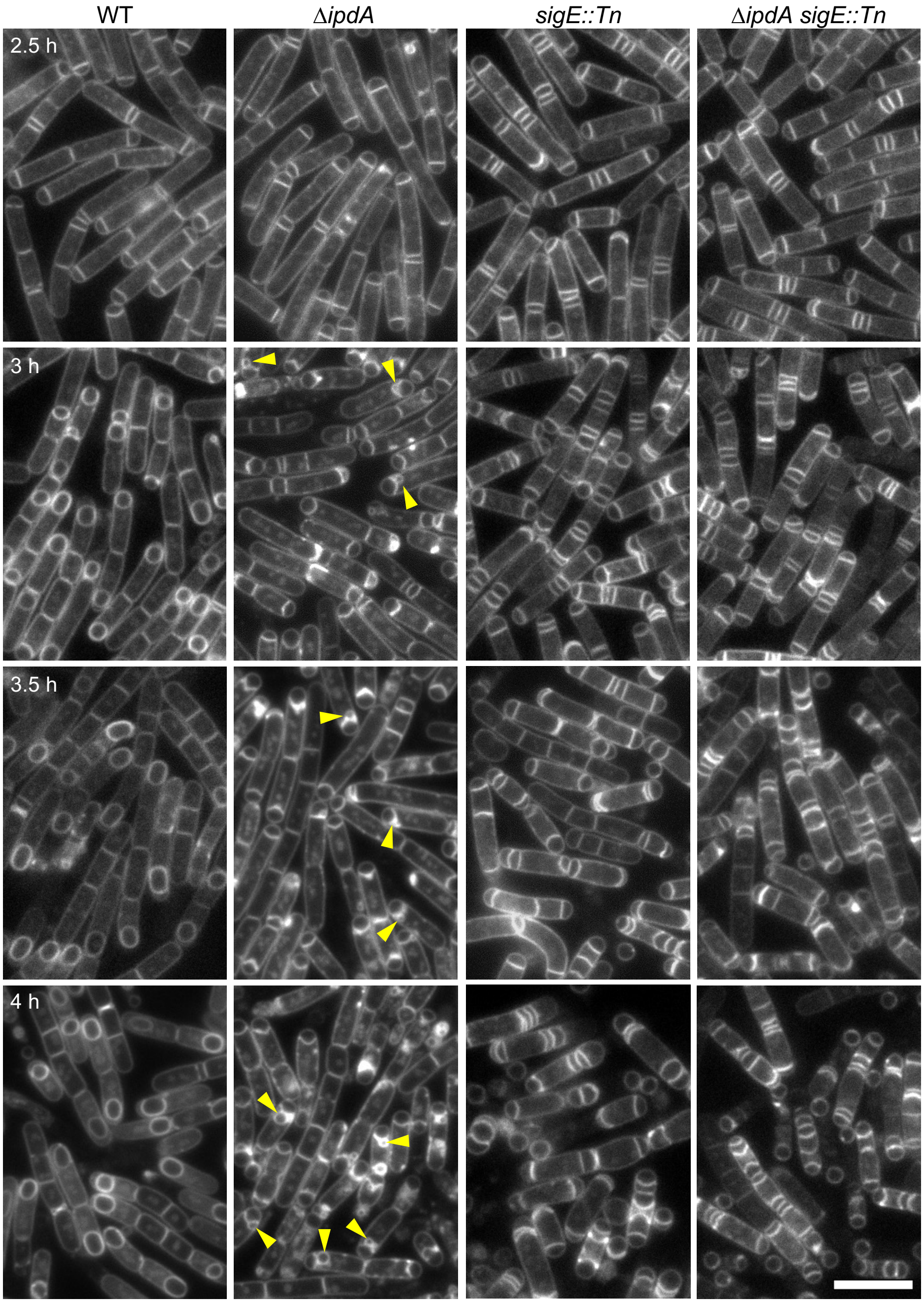

Figure H. Sporulating cells lacking *ipdA* and *sigE* lack septal bulges. Representative images of the indicated *B. anthracis* strains during a sporulation time course. Time after the onset of sporulation is indicated in the top left corner. In *B. subtilis,* sporulating cells lacking *sigE* do not initiate the mother-cell program of gene expression, including expression of the engulfment cell wall hydrolases. *B. anthracis* sporulating cells lacking *ipdA* and *sigE* do not form septal bulges, suggesting the engulfment hydrolases are responsible for these bulges. Yellow carets highlight septal bulges in the Δ*ipdA* mutant. Membranes were stained with TMA-DPH. Scale bar indicates 5 µm.

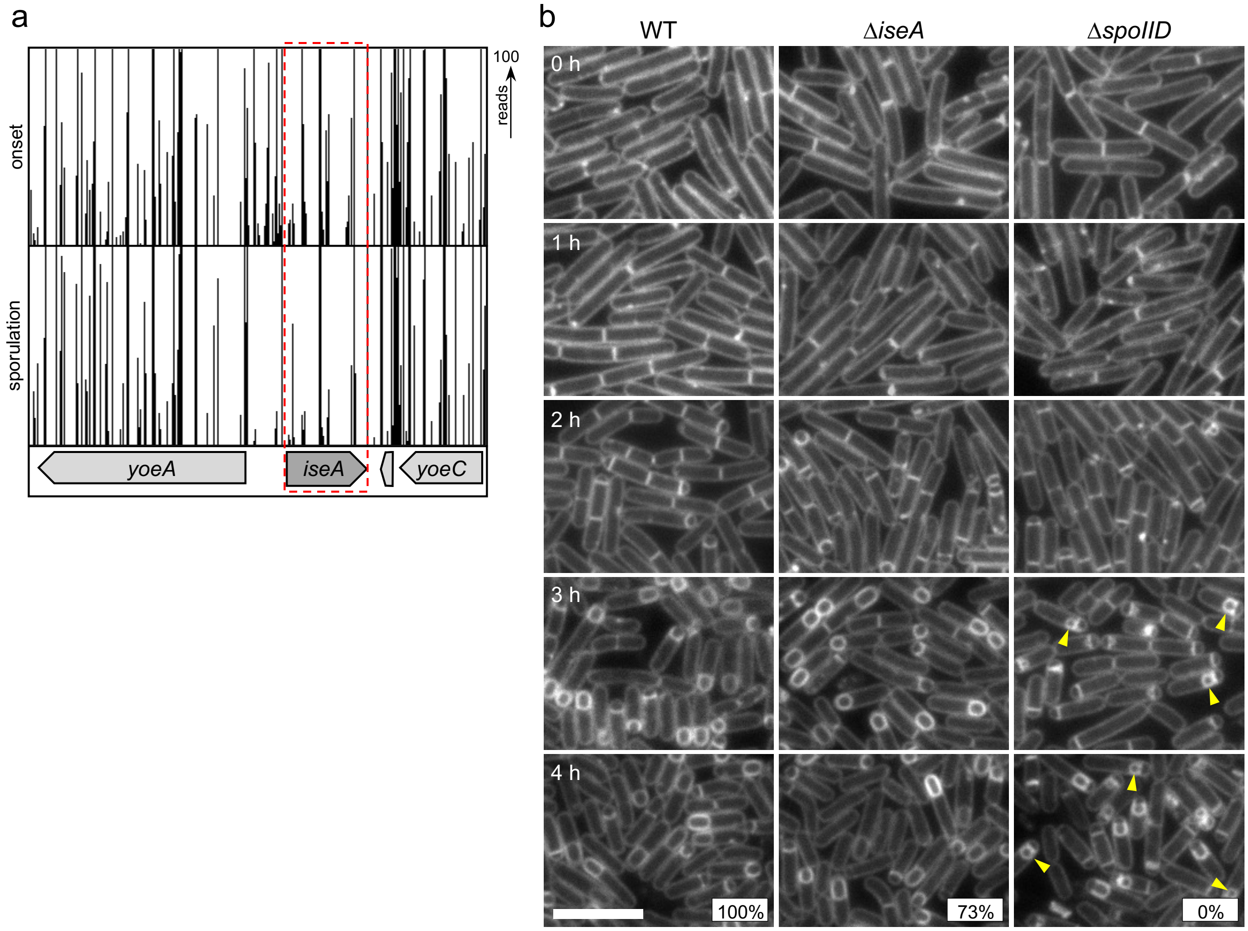

Figure I. IseA is not required for sporulation in *B. subtilis*. **(a)** Transposon-insertion profile of a region of the *B. subtilis* chromosome encompassing *iseA*. Transposon insertions in *iseA* are not underrepresented after sporulation, heat treatment, germination, and outgrowth compared to the onset of starvation. The Tn-seq data are from *Meeske et al 2016*. The *p*-value and fold-change in transposon insertions after sporulation compared to onset are 0.10 and 0.93, respectively. **(b)** Representative images of the indicated *B. subtilis* strains during a sporulation time course. Time after the onset of sporulation is indicated in the top left corner. Sporulating cells lacking IseA engulf normally and are indistinguishable from wild-type. The Δ*spoIID* mutant displays characteristic septal bulges (yellow carets). The sporulation efficiencies of the wild-type and the mutants are shown in the bottom right of the images at hour 4 of sporulation. Scale bar indicates 5 μm.

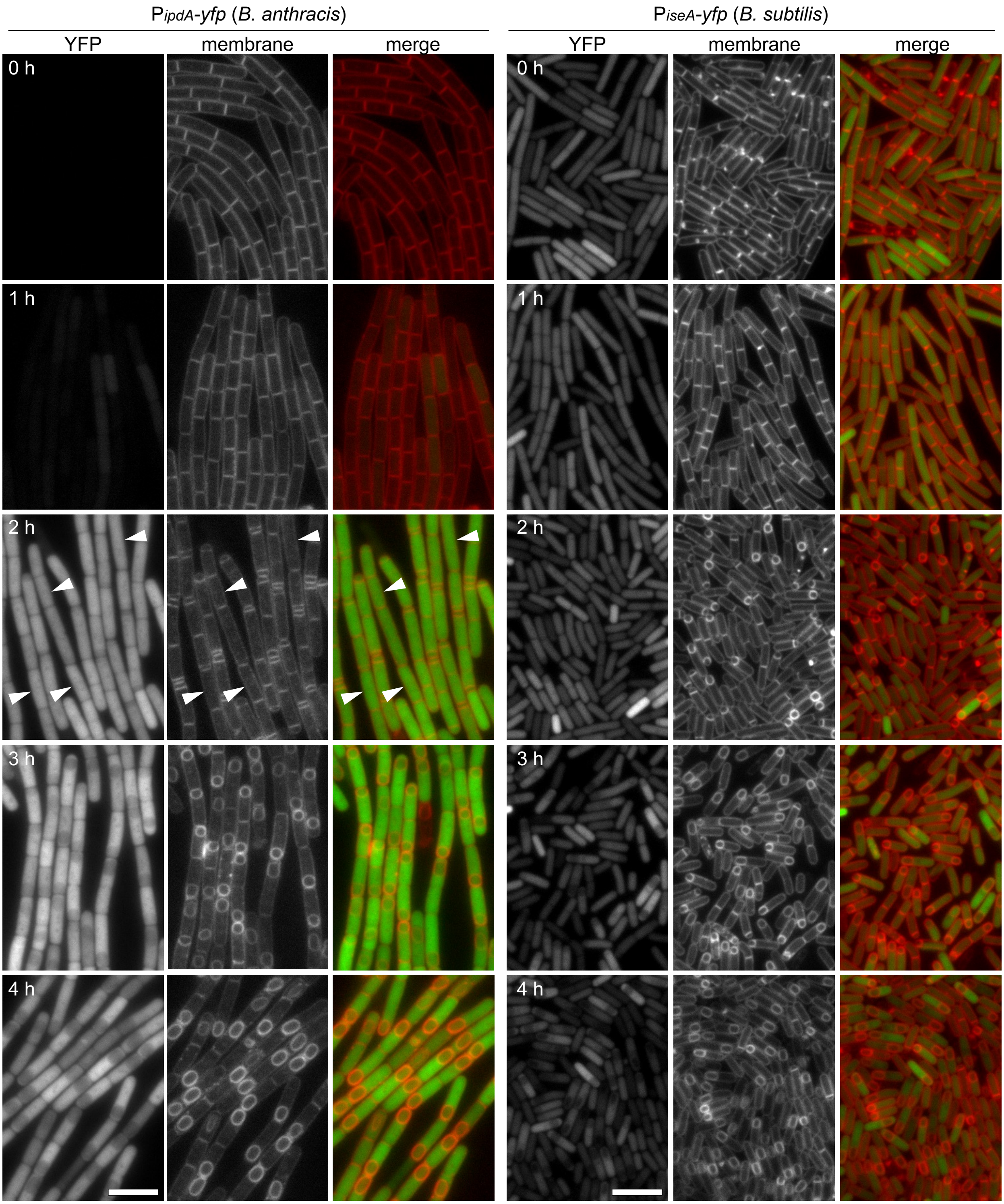

Figure J. Transcription of *B. anthracis ipdA* but not *B. subtilis iseA* is induced at the onset of sporulation. Representative fluorescence images of *B. anthracis* and *B. subtilis* strains harboring *ipdA* and *iseA* promoter fusions to *yfp* or *venus* (false-colored green) during a sporulation time-course. *B. anthracis* and *B. subtilis* were induced to sporulate by nutrient exhaustion and by resuspension, respectively. Membranes were stained with TMA-DPH. White carets highlight examples of sporulating cells that have induced P_{ipdA}-*yfp* prior to polar division. Scale bars indicate 5 μm.

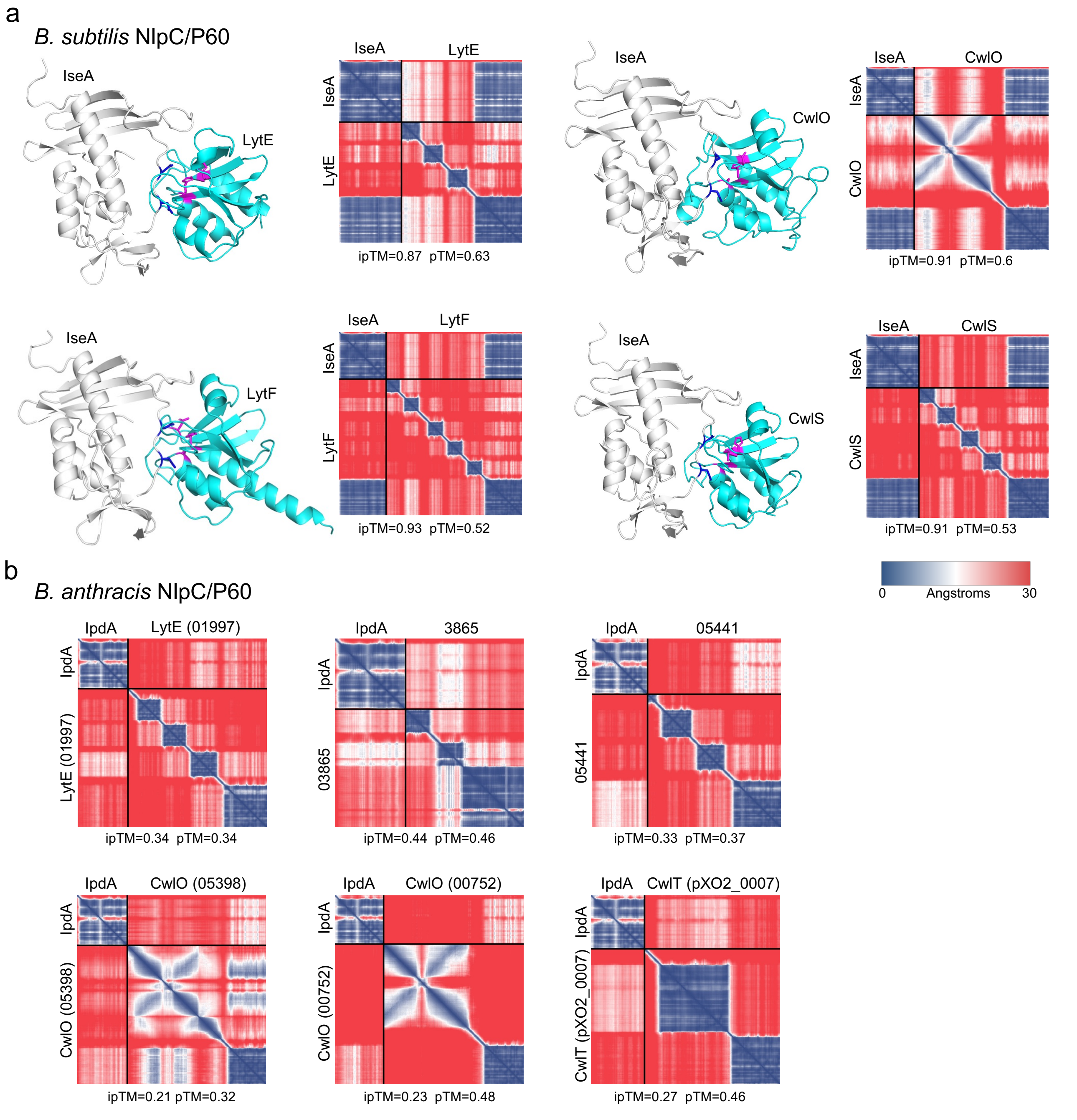

Figure K. AlphaFold3 predicts interactions between *B. subtilis* IseA and the NlpC/P60 catalytic domains of cell wall hydrolases. (a) Predicted structures of *B. subtilis* IseA (white) bound to LytE, LytF, CwlO, and CwlS (cyan). An unstructured loop in IseA is predicted to interact with the catalytic grooves of all four cell wall hydrolases. Only the catalytic domains of the four hydrolases are depicted. Catalytic residues are shown in magenta. Key interacting residues in IseA are colored dark blue. Predicted alignment error (pAE) plots, as well as predicted template modeling (pTM) and interface predicted template modeling (ipTM) scores, are shown for each prediction. **(b)** IpdA is not predicted to interact with any of the NlpC/P60 domain-containing proteins in *B. anthracis*. pAE plots, and pTM and ipTM scores for each prediction are shown.

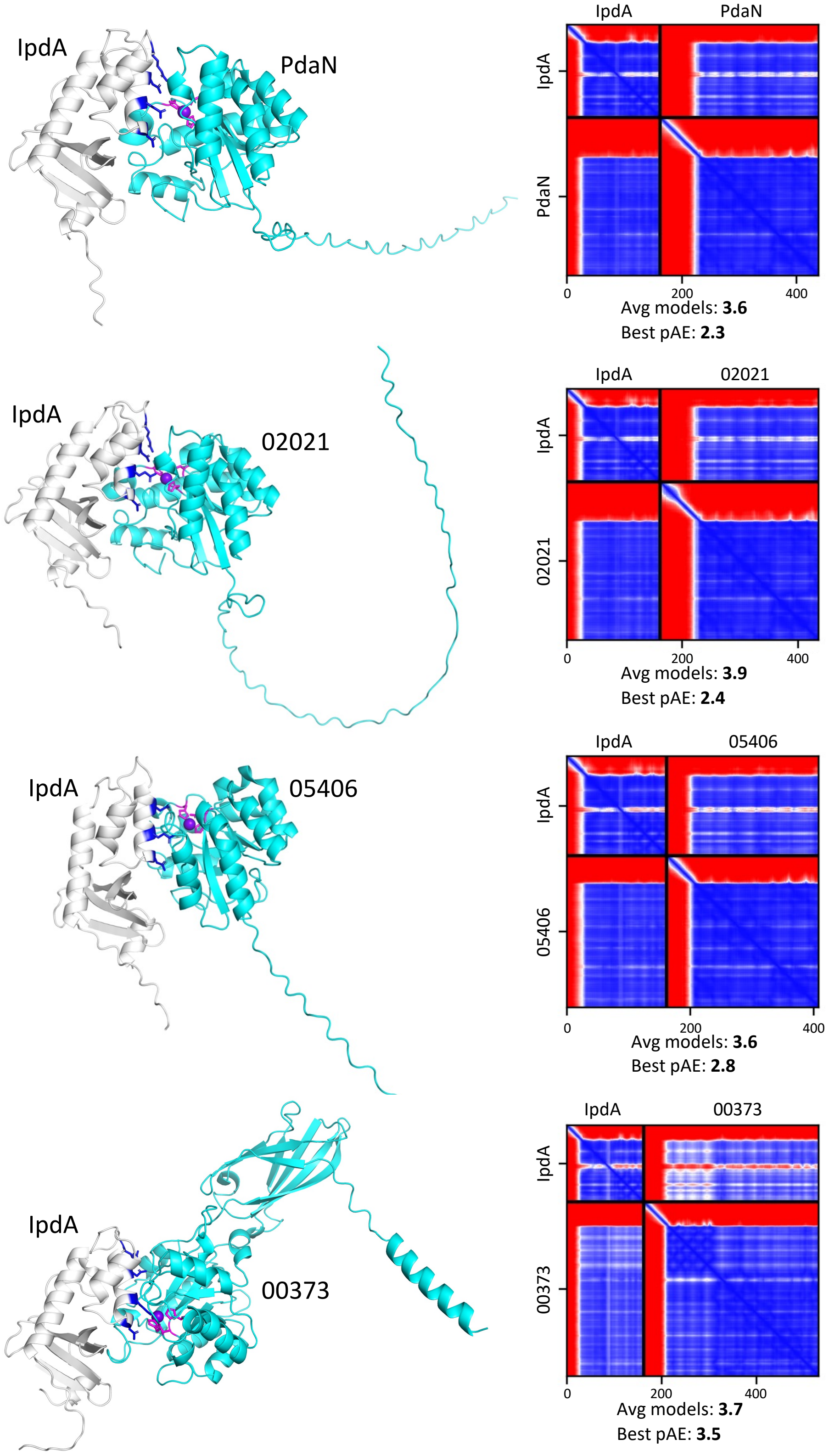

Figure L. AlphaFold-predicted structures of *B. anthracis* IpdA (white) bound to four polysaccharide deacetylases (cyan). The catalytic residues of the deacetylase (magenta) and Zn^{2+} ligand (purple) are shown. Predicted alignment error (pAE) plots, average model scores, and lowest pAE from the AlphaFold2-multimer screen are shown on the right.

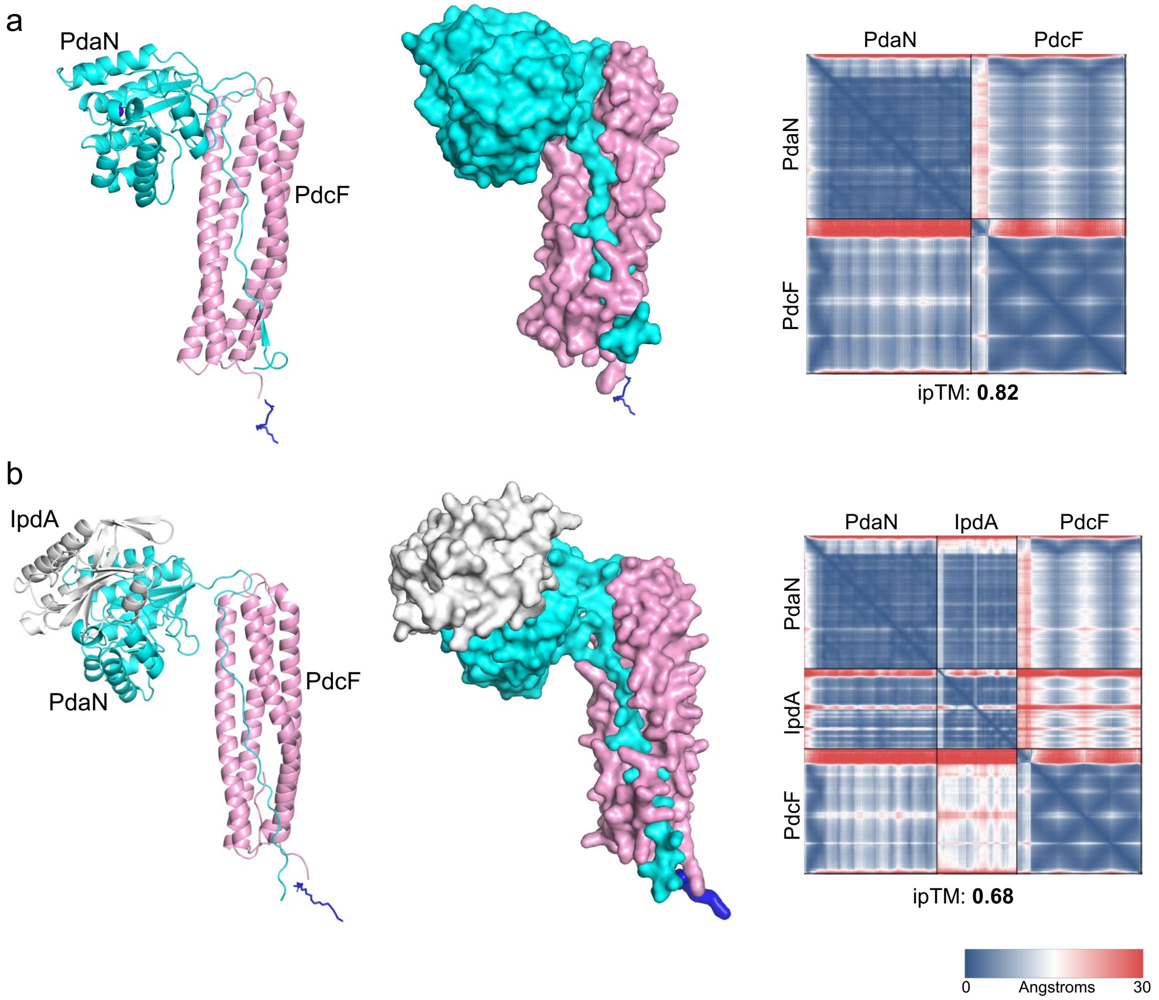

Figure M. Predicted structures of *B. anthracis* PdaN bound to PdcF with and without IpdA. (a) AlphaFold3-predicted complex of PdaN (cyan) and PdcF (pink). Cartoon and surface models are shown. The predicted lipidated N-terminus of PdcF is in dark blue. pAE plot and ipTM score are shown on the right. **(b)** AlphaFold3-predicted complex of PdaN (cyan), PdcF (pink), and IpdA (white). Cartoon and surface models are shown. pAE plot and ipTM score are shown on the right.

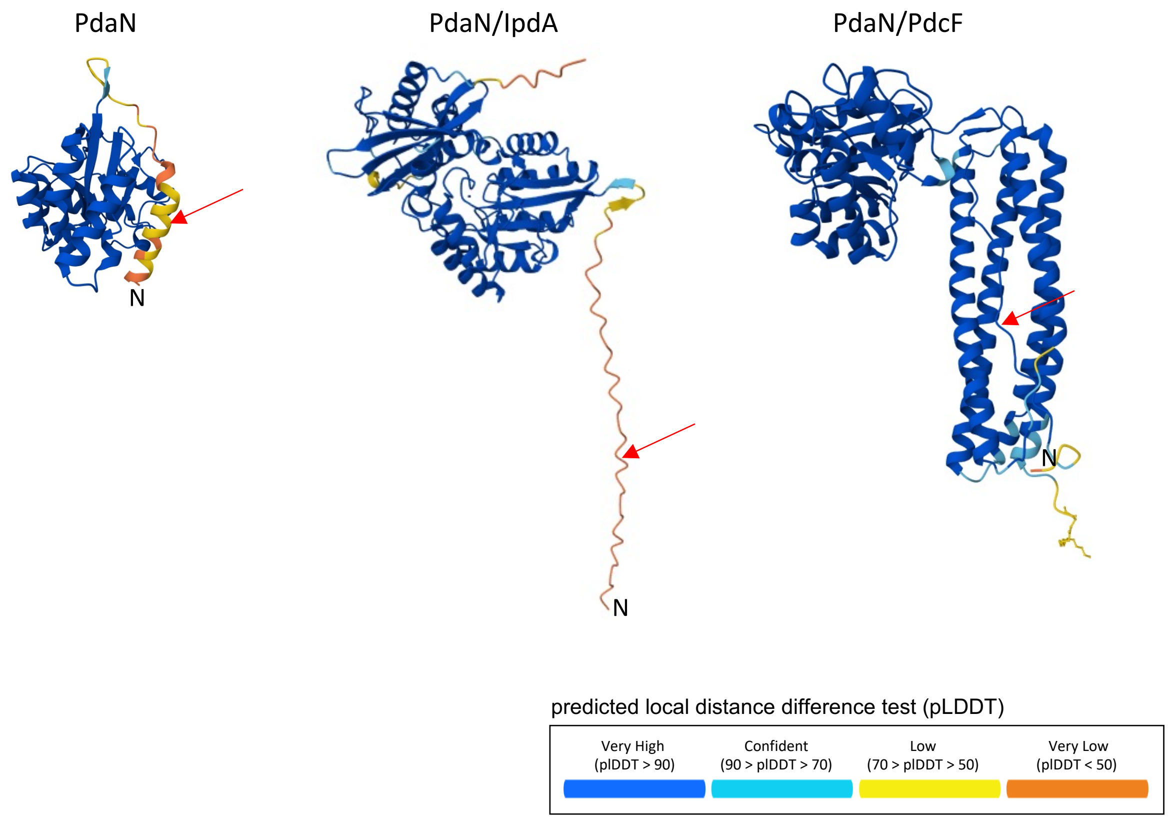

Figure N. PdaN's N-terminus transitions from low to high per-residue local confidence in the presence of PdcF. AlphaFold3-predicted structures of PdaN, the PdaN/IpdA complex, and the PdaN/PdcF complex. The structures are colored based on a per-atom confidence estimates. Dark blue indicates very high confidence, while yellow and orange indicate low and very low confidence, respectively. The per-residue local confidence of the N-terminus of PdaN is low or very low in the first two models. However, the per-residue local confidence of this region becomes high in the predicted complex with PdcF. The increase in local confidence provides additional support for the predicted PdaN/PdcF model.

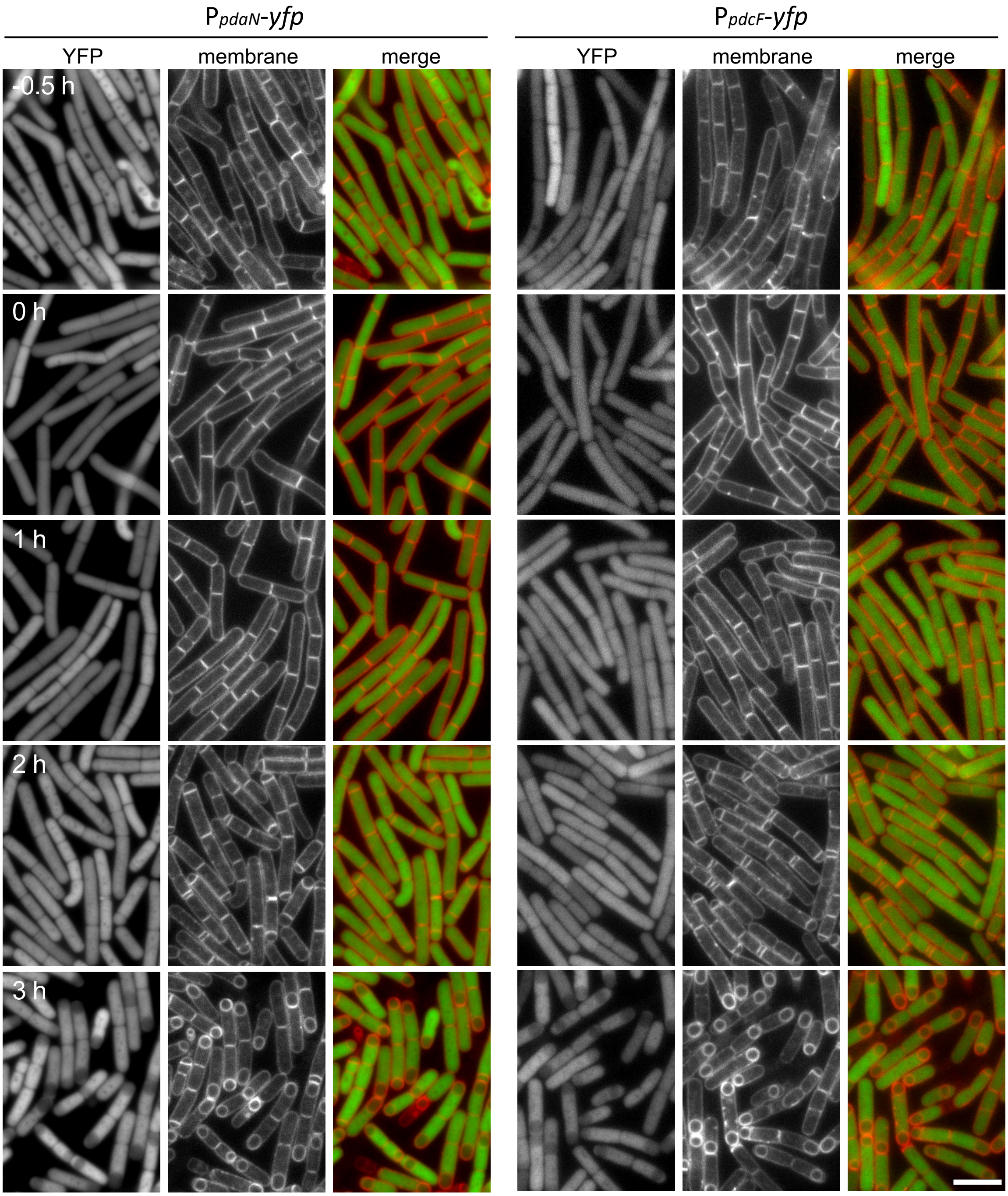

Figure O. The *pdaN* and *pdcF* genes are expressed prior to the onset of starvation and throughout sporulation. Representative fluorescence images of *B. anthracis* strains harboring *pdaN* and *pdcF* promoter fusions to *yfp* during a sporulation time-course. Time (in hours) before and after the onset of sporulation are indicated in the top left corner. Membranes were stained with TMA-DPH. Scale bar indicates 5µm.

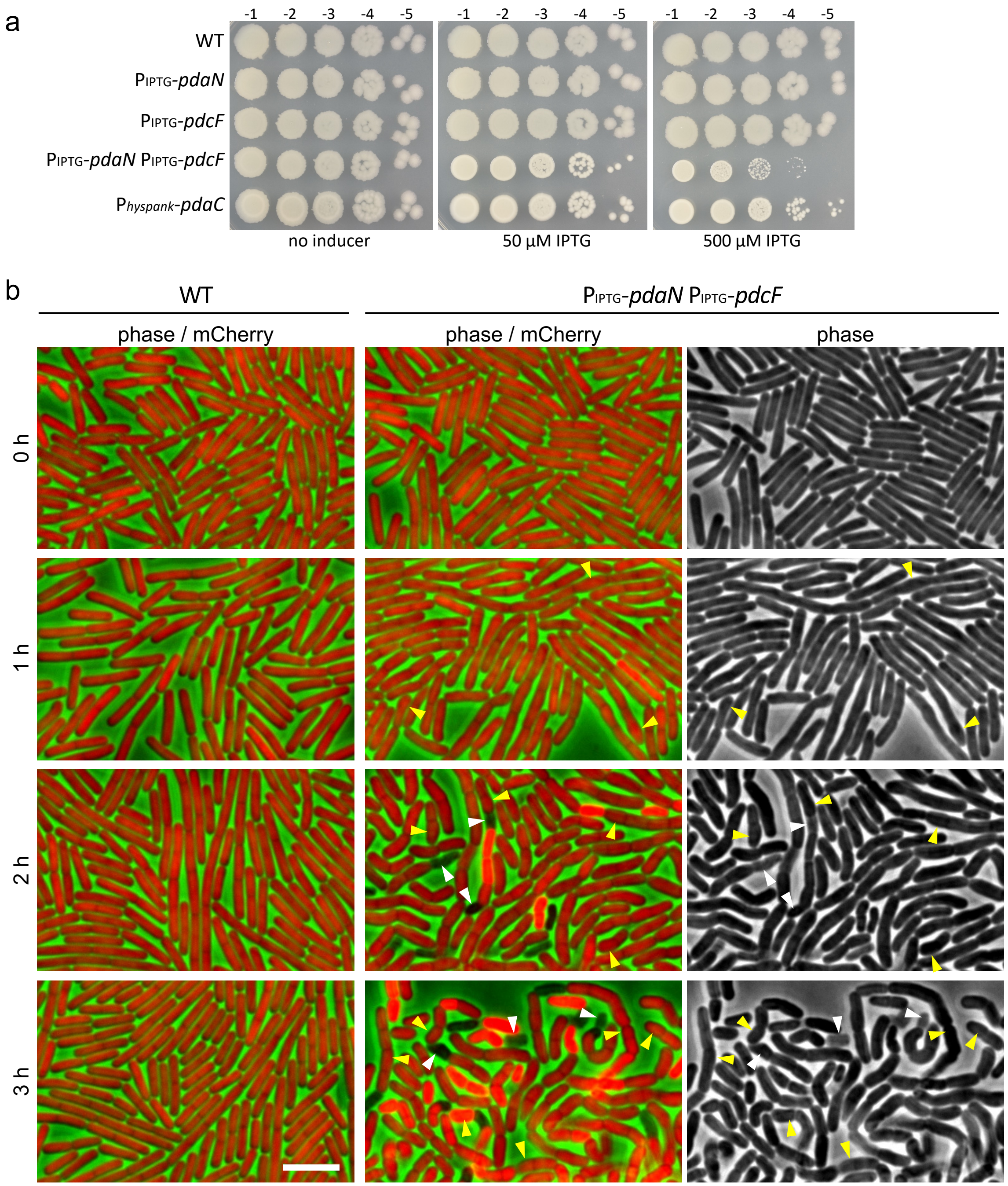

Figure P. Expression of *B. anthracis pdaN* and *pdcF* in *B. subtilis* inhibits cell elongation. (a) Photographs of spot-dilutions of the indicated *B. subtilis* strains on LB agar plates. 10-fold serial dilutions of the indicated strains were spotted on LB agar supplemented without or with 50 µM or 500 µM IPTG. The *pdaN* and *pdcF* genes were fused to the P_{spank} (P_{IPTG}) promoter. The *B. subtilis pdaC* gene was fused to the $P_{hyperspank}$ ($P_{hyspank}$) promoter. The $P_{hyperspank}$ promoter is ~7-fold stronger than the P_{spank} promoter. **(b)** Representative fluorescence and phase-contrast images of exponentially growing cells before and after addition of IPTG. Exponentially growing cultures of the indicated strains expressing cytoplasmic mCherry (red) were analyzed at the indicated times after IPTG (500 µM, final) addition. Short, chubby cells (yellow carets) and lysed cells (white carets) are characteristic of inhibition of cell wall elongation. Scale bar indicates 5 µm.

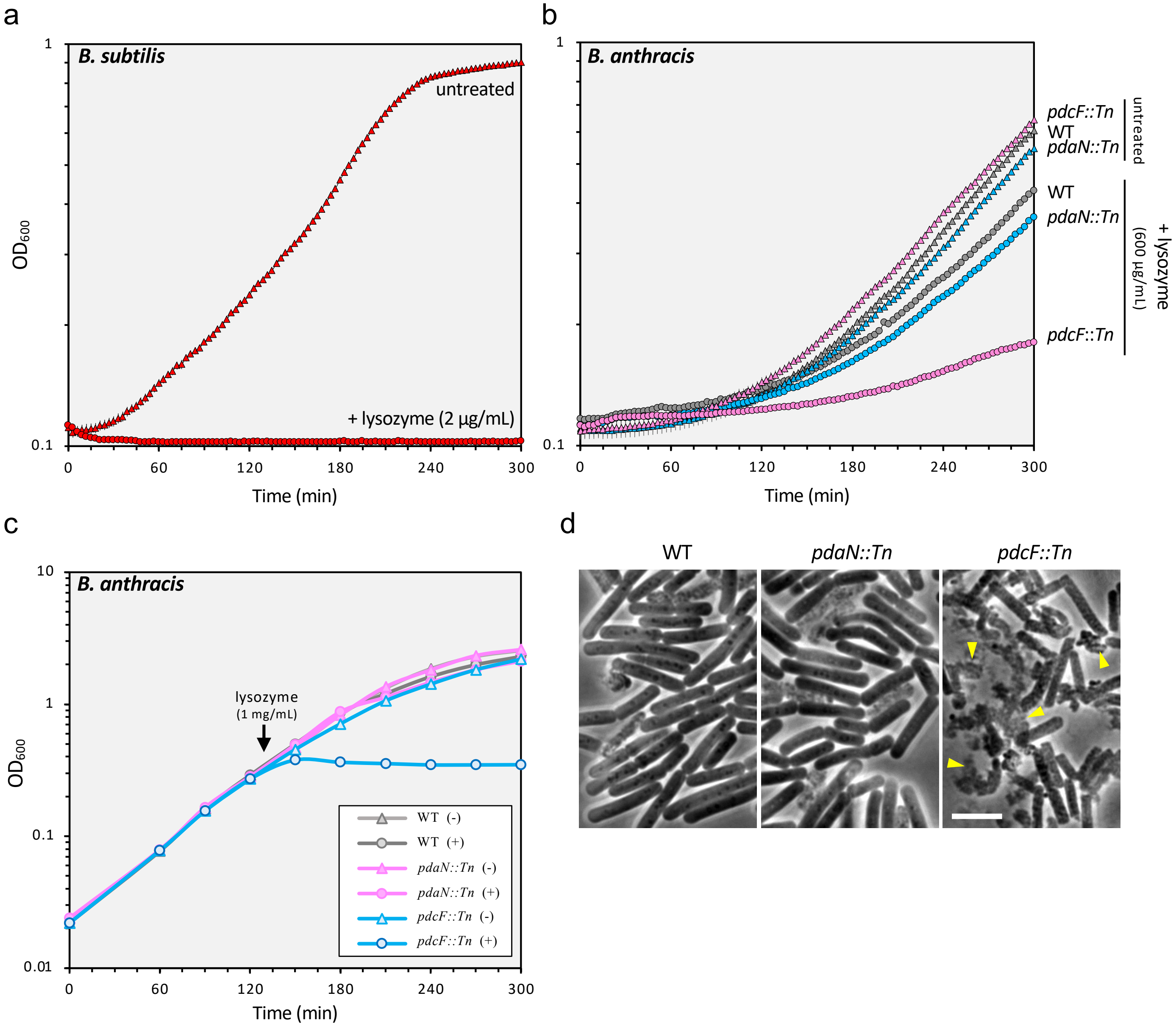

Figure Q. *B. anthracis* cells lacking *pdcF* are more sensitive to lysozyme. Growth curves of *B. subtilis* (**a**) and *B. anthracis* (**b**) chronically exposed to lysozyme (2 µg/mL for *B. subtilis;* 600 µg/mL for *B. anthracis*). The indicated strains were grown in sporulation media until mid-exponential phase and back-diluted into fresh media with and without the indicated concentrations of lysozyme. OD_{600} was recorded every 3 min for 5 h. Growth curves show the mean of three technical replicates ± standard deviation. **(c)** Growth curve of the indicated *B. anthracis* strains before and after acute exposure to 1 mg/mL lysozyme (arrow). The indicated strains were grown in sporulation media until mid-exponential phase and back-diluted to an OD_{600} of 0.05. When the cultures reached an OD_{600} ~0.3, they were divided in two. One subculture was exposed to lysozyme (1 mg/mL, final) and the other was left untreated. OD_{600} was recorded every 30 min for 3 h. **(d)** Representative phase-contrast images of the indicated strains in (c) 3 h after addition of lysozyme (1 mg/mL, final). Yellow carets highlight lysed cells. Scale bar indicates 5µm. Representative growth curves from one of two biological replicates are shown in panels a, b and c, with the corresponding underlying data available in S1 Data.

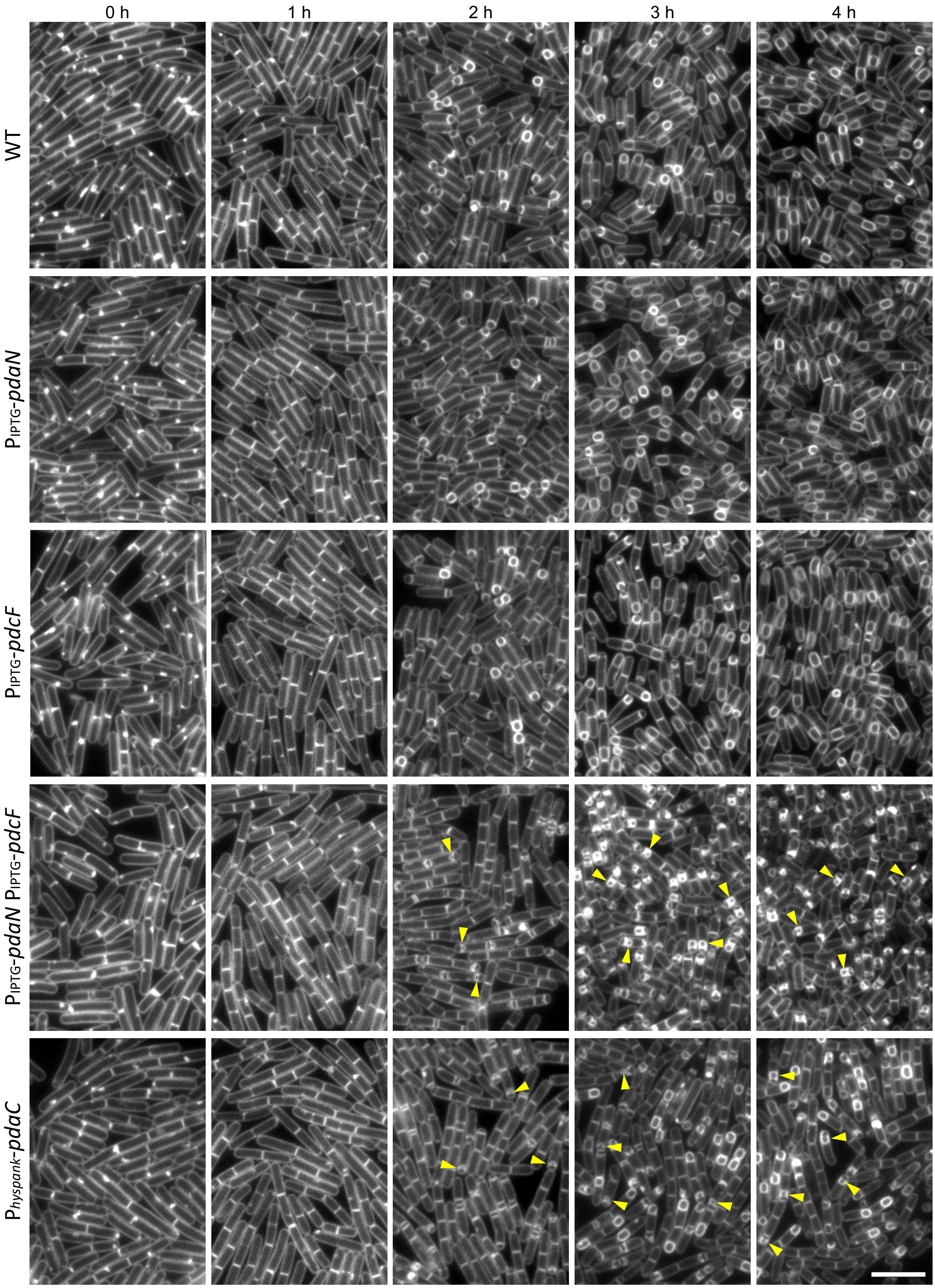

Figure R. Expression of *B. anthracis pdaN* and *pdcF* in *B. subtilis* causes septal membrane bulging during sporulation. Representative images of the indicated strains during a sporulation time course. Sporulation was induced by the resuspension method, and IPTG (500 µM, final) was added to the cultures at the time of resuspension (0 h). Cells were examined by fluorescence microscopy at the indicated time points. Septal bulges (yellow carets) are highlighted. Scale bar indicates 5 µm. Expression of *B. subtilis* PdaC, a membrane-anchored MurNAc deacetylase that is not normally produced during sporulation, was included for comparison.

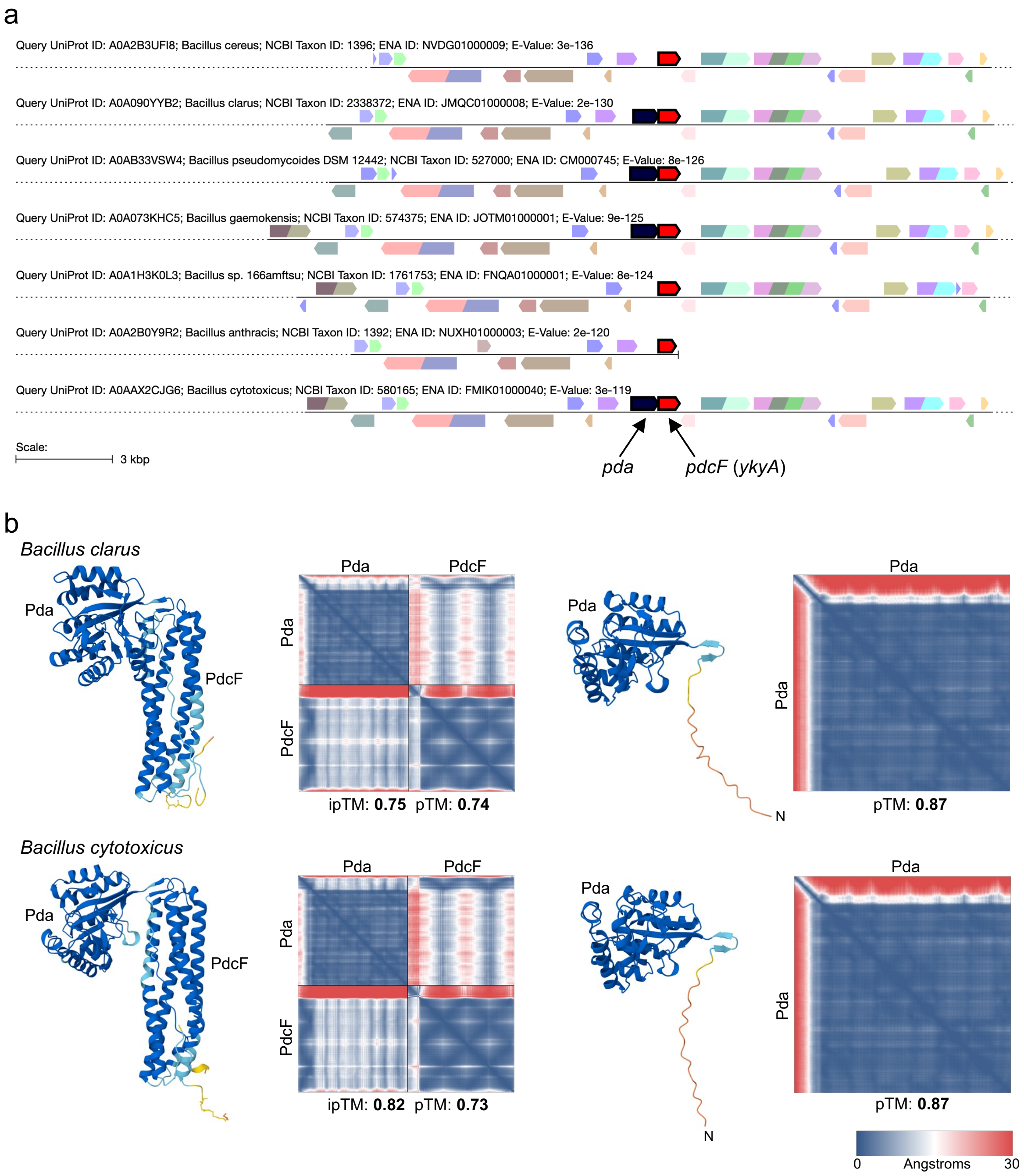

Figure S. Genes encoding secreted polysaccharide deacetylases can be found adjacent to homologs of PdcF. (a) Gene neighborhood analysis using PdcF as the query identifies a subset of *Bacilli* in which genes encoding a putative polysaccharide deacetylase (*pda*) and a PdcF homolog (*pdcF*, *ykyA*) are adjacent to each other. **(b)** AlphaFold3 predicts that the putative deacetylases (Pda) and their putative PdcF co-factors form a complex. Predicted structures are colored based on per-atom confidence estimates. Dark blue indicates very high confidence, and light blue indicates confident. The pAE plots and ipTM and pTM scores are shown to the right. The AlphaFold3-predicted structures of the deacetylases are also shown. The predicted structures are colored based on per-atom confidence estimates. The N-terminal domains of both are orange indicating very low per atom confidence estimates.

a

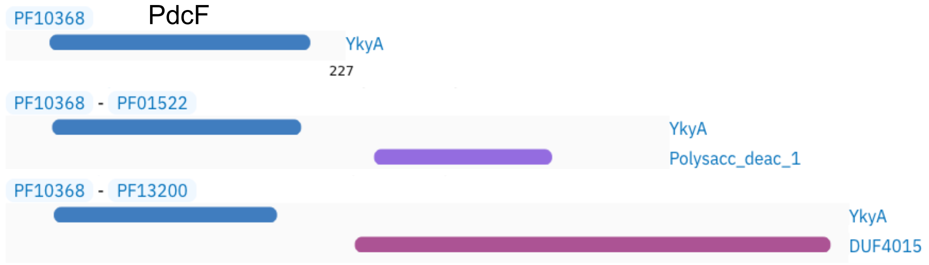

b

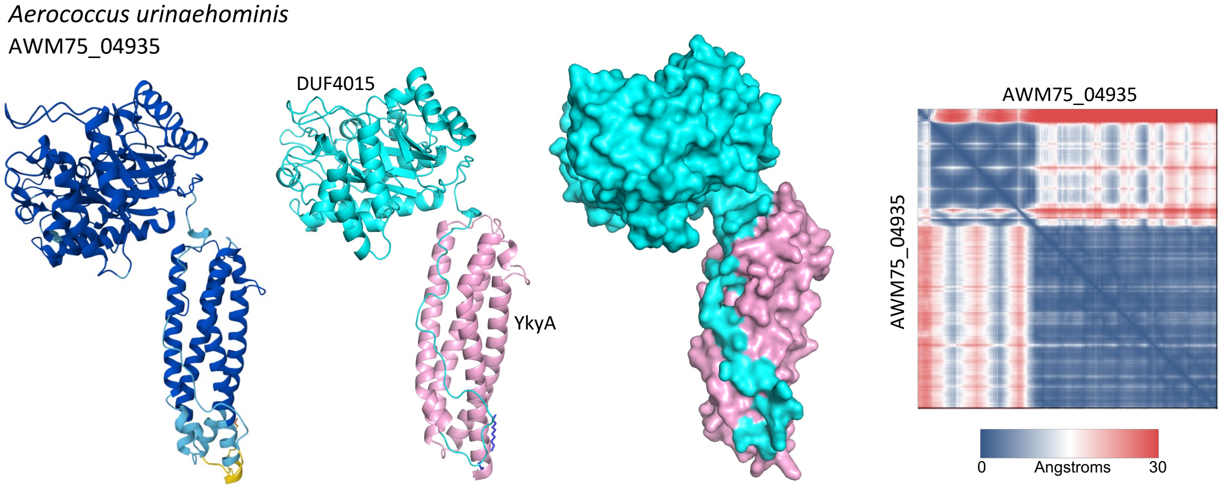

Figure T. The YkyA domain is fused to different enzymatic domains. (a) Schematic from Pfam highlighting examples of stand-alone YkyA domains like PdcF and gene fusions with polysaccharide deacetylase domains and DUF4015 domains. **(b)** AlphaFold3-predicted structure of AWM75_04935 lipoprotein from *A. urinaehominis*. The structure on the left is colored based on a per-atom confidence estimates. Dark blue indicates very high confidence, and light blue indicates confident. The two adjacent structures are colored using the same scheme as PdaN (cyan) and PdcF (pink). The YkyA domain is fused to the DUF4015 domain in this protein. The unstructured region that links the two domains interacts with the four-helix bundle, similar to PdaN and PdcF. The pAE plot is shown on the right.

Table A. List of *Bacillus* strains used in this study.

***B. anthracis* strain**	**Genotype**	**Source**	**Figure(s)**
BaR1	Wild-type (*B. anthracis* Sterne 9131)	James Kirby	2, 4F, 5H, 5J, G, H, Q
BaR6	Wild-type (*B. anthracis* Sterne NR-9401 pXO1^{+} [*cya^{-} pagA^{-} lef^{-}*] pXO2^{-})	BEI Resources	4B, 5B, 5I
BaR9	395/396::P$_{spoIID}$-*optRBS-cfp* (*kan*)<br>380/381::P$_{spoIIQ}$-optRBS-*yfp* (*cat*)	This work	2B, 3, 4A, D, E, Fa
BaR186	395/396::P$_{spoIID}$-*optRBS-cfp* (*kan*)<br>380/381::P$_{spoIIQ}$-*optRBS-yfp* (*cat*)<br>*spoIIE::Tn (spec)*	This work	2, D, E
BaR180	395/396::P$_{spoIID}$-*optRBS-cfp* (*kan*)<br>380/381::P$_{spoIIQ}$-*optRBS-yfp* (*cat*)<br>*spoIIR::Tn (spec)*	This work	2, D, E
BaR185	395/396::P$_{spoIID}$-*optRBS-cfp* (*kan*)<br>380/381::P$_{spoIIQ}$-*optRBS-yfp* (*cat*)<br>*spoIID::Tn (spec)*	This work	2, D, E
BaR187	395/396::P$_{spoIID}$-*optRBS-cfp* (*kan*)<br>380/381::P$_{spoIIQ}$-*optRBS-yfp* (*cat*)<br>*spoIIQ::Tn (spec)*	This work	2, D, E
BaR181	395/396::P$_{spoIID}$-*optRBS-cfp* (*kan*)<br>380/381::P$_{spoIIQ}$-*optRBS-yfp* (*cat*)<br>*spoIIIAE::Tn (spec)*	This work	2, D, E
BaR222	395/396::P$_{spoIID}$-*optRBS-cfp* (*kan*)<br>380/381::P$_{spoIIQ}$-*optRBS-yfp* (*cat*)<br>*yyaC::Tn (spec)*	This work	3, Fa
BaR226	395/396::P$_{spoIID}$-*optRBS-cfp* (*kan*)<br>380/381::P$_{spoIIQ}$-*optRBS-yfp* (*cat*)<br>*yqfT::Tn (spec)*	This work	3, Fa
BaR248	395/396::P$_{spoIID}$-*optRBS-cfp* (*kan*)<br>380/381::P$_{spoIIQ}$-*optRBS-yfp* (*cat*)<br>*yuzB::Tn (spec)*	This work	3, Fa
BaR225	395/396::P$_{spoIID}$-*optRBS-cfp* (*kan*)<br>380/381::P$_{spoIIQ}$-*optRBS-yfp* (*cat*)<br>*yhfN::Tn (spec)*	This work	3, Fb
BaR251	395/396::P$_{spoIID}$-*optRBS-cfp* (*kan*)<br>380/381::P$_{spoIIQ}$-*optRBS-yfp* (*cat*)<br>*ytxC::Tn (spec)*	This work	3, Fb
BaR250	395/396::P$_{spoIID}$-*optRBS-cfp* (*kan*)<br>380/381::P$_{spoIIQ}$-*optRBS-yfp* (*cat*)<br>*ysxE::Tn (spec)*	This work	3, Fb
BaR124	*murAB::Tn (spec)*	This work	G
BaR498	380/381::P$_{spoIIQ}$-*optRBS-yfp* (*kan*)<br>*ipdA::Tn (spec)*	This work	4A
BaR490	395/396::*ipdA* (*kan*)<br>*ipdA::Tn (spec)*	This work	4A
BaR469	395/396::P$_{ipdA}$-*optRBS-yfp* (*kan*)	This work	4E, J
BaR244	Δ*ipdA::kan*	This work	4F, 5H, H
BaR12	*sigE::Tn (spec)*	This work	4F, H
BaR432	Δ*ipdA::kan*<br>*sigE::Tn (spec)*	This work	4F, H
BaR419	Δ*ipdA::kan*<br>*pdaN::Tn (spec)*	This work	5H
BaR499	Δ*ipdA::kan*<br>*pdcF::Tn (spec)*	This work	5H
BaR491	395/396::*ipdA-his$_{6}$* (*kan*)<br>*ipdA::Tn (spec)*	This work	5J
BaR507	395/396::*ipdA*$^{N73A, Q76A, D80A}$-*his$_{6}$* (*kan*)<br>*ipdA::Tn (spec)*	This work	5J
BaR487	395/396::P$_{pdaN}$-*optRBS-yfp* (*kan*)	This work	O

BaR488	395/396:: P_{pdcF}-*optRBS-yfp* (*kan*)	This work	O
BaR402	*pdaN::Tn (spec)*	This work	Q
BaR403	*pdcF::Tn (spec)*	This work	Q

***B. subtilis* strain**	**Genotype**	**Source**	**Figure(s)**
PY79	Prototrophic 168 derivative	Youngman *et al.,* 1983 (1)	2, 4C, 5K, I, P, Q, R
BDG169	Δ*iseA::erm*	Laboratory stock	4C, I
BDR1360	Δ*spoIID::spec*	Laboratory stock	4C, I
BDG110	*amyE::*P_{iseA}-*optRBS-venus* (*cat*)	Dobihal *et al.,* 2019 (2)	J
BDR4539	*amyE::*P_{spank}-*optRBS-pdcF*(*Ba*) (*spec*)	This work	P, R
BDR4547	*yvbJ::*P_{spank}-*optRBS-pdaN*(*Ba*) (*kan*)	This work	P, R
BDR4548	*amyE::*P_{spank}-*optRBS-pdcF*(*Ba*) (*spec*), *yvbJ::*P_{spank}-*optRBS-pdaN*(*Ba*) (*kan*)	This work	5K, P, R
BDR4549	*sacA::*P_{veg}-*mCherry* (*tet*)	This work	5G, P
BDR4550	*amyE::*P_{spank}-*optRBS-pdcF*(*Ba*) (*spec*), *yvbJ::*P_{spank}-*optRBS-pdaN*(*Ba*) (*kan*), *sacA::*P_{veg}-*mCherry* (*tet*)	This work	5G, P
BDG810	*yvbJ::*$P_{hyperspank}$-*optRBS-pdaC* (*spec*)	Dobihal *et al.,* 2022 (3)	P, R

Supplemental References:

1. Youngman PJ, Perkins JB, Losick R. Genetic transposition and insertional mutagenesis in *Bacillus subtilis* with *Streptococcus faecalis* transposon Tn917. Proc Natl Acad Sci USA. 1983; 80(8):2305–9. PMID: 6300908; PubMed Central PMCID: PMCPMC393808.

2. Dobihal GS, Brunet YR, Flores-Kim J, Rudner DZ. Homeostatic control of cell wall hydrolysis by the WalRK two-component signaling pathway in *Bacillus subtilis*. Elife. 2019; 8. Epub 20191206. https://doi.org/10.7554/eLife.52088 PMID: 31808740; PubMed Central PMCID: PMC7299342

3. Dobihal GS, Flores-Kim J, Roney IJ, Wang X, Rudner DZ. The WalR-WalK signaling pathway modulates the activities of both CwlO and LytE through control of the peptidoglycan deacetylase PdaC in *Bacillus subtilis*. J Bacteriol. 2022; https://doi.org/10.1128/jb.00533-21. PMID: 34871030; PubMed Central PMCID: PMC8846395

Table B. List of plasmids used in this study.

Plasmid	Description	Source
pFR38	Himar1C9 IR-Spec (*amp*, *erm, spec*)	This work
pFR50	pMiniMAD_P$_{veg}$-*mCherry* (*amp*, *erm*)	This work
pFR76	*yvbJ::*P$_{spank}$-*optRBS-pdaN*(*Ba*) (*amp*, *kan*)	This work
pFR77	*amyE::*P$_{spank}$-*optRBS-pdcF*(*Ba*) (*amp*, *spec*)	This work
pBaR5	pMiniMAD_P$_{veg}$-*mCherry* – 395/396 (*amp, erm, kan*)	This work
pBaR8	pMiniMAD_P$_{veg}$-*mCherry* – 380/381 (*amp, erm, cat*)	This work
pBaR9	pMiniMAD_P$_{veg}$-*mCherry* – 395/396::P$_{spoIID}$-*optRBS-cfp* (*amp, erm, kan*)	This work
pBaR10	pMiniMAD_P$_{veg}$-*mCherry* – 380/381::P$_{spoIIQ}$-*optRBS-yfp* (*amp, erm cat*)	This work
pBaR20	pMiniMAD_P$_{veg}$-*mCherry* – 395/396::P$_{ipdA}$-*optRBS-yfp* (*amp, erm, kan*)	This work
pBaR28	pMiniMAD_P$_{veg}$-*mCherry* – Δ*ipdA*::*kan* (*amp, erm, kan*)	This work
pBaR36	pMiniMAD_P$_{veg}$-*mCherry* – 395/396::*ipdA* (*amp, erm, kan*)	This work
pBaR39	pMiniMAD_P$_{veg}$-*mCherry* – 395/396::P$_{pdaN}$-*optRBS-yfp* (*amp, erm, kan*)	This work
pBaR40	pMiniMAD_P$_{veg}$-*mCherry* – 395/396::P$_{pdcF}$-*optRBS-yfp* (*amp, erm, kan*)	This work
pBaR44	pMiniMAD_P$_{veg}$-*mCherry* – 395/396::*ipdA-his$_6$* (*amp, erm, kan*)	This work
pBaR47	pMiniMAD_P$_{veg}$-*mCherry* – 395/396::*ipdA*(N73A, Q76A, D80A)-*his$_6$* (*amp, erm, kan*)	This work

Table C. List of oligonucleotide primers used in this study

Primer	Sequence	Use
oFR295	gccGGATCCatgatttgggatattggcgga	pBaR5 (upstream region)
oFR296	gccGGGCCCccggtgtcattataatgattg	
oFR297	gccGGGCCCcagttcatgtgacatgaacgt	pBaR5 (downstream region)
oFR298	gccGTCGACggtagactactacctatcaaa	
oFR299	gccGGATCCgtgacaaaaggagaactaggc	pBaR8 (upstream region)
oFR300	gccGGGCCCgcaactccaaatatatcagcg	
oFR301	gccGGGCCCggagttgcaatcgatatgttc	pBaR8 (downstream region)
oFR302	gccGTCGACgttgcctcttccgcgggattc	
oFR309	gccGGTACCctgatcttcacgcttaattga	pBaR9 (*spoIID* promoter)
oFR310	gccGAATTCtataaaaaagctttactccattca	
oFR311	gccGAATTCacataaggaggaactactatg	*optRBS-cfp*
oFR312	gccGCTAGCttacttataaagttcgtccat	
oFR314	gccGGTACCcgctgggtttagtagtattag	pBaR10 (*spoIIQ* promoter)
oFR315	gccGAATTCagcaatcattttgaacaatat	
oFR320	gccACTAGTttatttgtatagttcatccat	*optRBS-yfp* (reverse)
oFR465	gccGGTACCctggtgagatggtacgaaca	pBaR20 (*ipdA* promoter)
oFR466	gccGAATTCttttaagaaaacttgtctttgtag	
oFR527	gccGTCGACcgattaataaagtcggaacaggta	pBaR28 (upstream region)
oFR528	gccGGGCCCtccatatttcctcatttttcaccttc	
oFR531	gccGGGCCCgccactagtaggttacaacaattataaagtgaaact	pBaR28 (downstream region)
oFR532	gccGGATCCgcagtataagacgcagaaatacga	
oFR554	gccGGTACCctggtgagatggtacgaacat	pBaR36 (*ipdA* gene)
oFR555	gccGCTAGCgtatacatccgattgtggcg	
oFR564	gccGGTACCcctaccgtaaatatgctcgtc	pBaR39 (*pdaN* promoter)
oFR565	gccGAATTCtataaaagttctactctactatattagc	
oFR566	gccGGTACCggtaaatgattactgttgttc	pBaR40 (*pdcF* promoter)
oFR567	gccAAGCTTcttatatacgatcatcgtgt	
oFR568	gccAAGCTTacataaggaggaactactatg	*optRBS-yfp* (forward)
oFR587	gccGCTAGCttagtggtgatggtgatgatgtaattgttgtaaccttttttatttc	*ipdA* gene (C-terminal his_6)
oFR590	caccatattttacagaggctttccttgcagtatttacagctgaaaatagtagaagtg	pBaR47 (site-directed mutagenesis)
oFR591	cacttctactattttcagctgtaaatactgcaaggaaagcctctgtaaaatatggtg	
oFR618	ggataacaattaagcttgtcccgggtaactagtacataaggaggaactactatgcacaatggaattcgaatgac	pFR76 (*pdaN* gene, isothermal assembly)
oFR619	cgaattagcttgcatgcgagctagcatctgcagggagttgagtcgtagtatatgagg	

Capital letters indicate restriction enzyme recognition sites, and underlined bases indicate mutations.
