# Supplementary material for: Identification of sporulation genes in Bacillus anthracis highlights similarities and significant differences with Bacillus subtilis
Source: PLoS Biol. 2025 Dec 12;23(12):e3003521. doi: 10.1371/journal.pbio.3003521 (PMC12700454; doi:10.1371/journal.pbio.3003521)
Supplement: Attachment — Submitted filename: Point-by-point responses (Ramirez et al).pdf [file pbio.3003521.s014.pdf]

REVIEWERS' COMMENTS:

Reviewer #1:
The manuscript by Ramírez-Guadiana et al. describes the use of TnSeq to identify genes required for spore formation, germination, and/or outgrowth (defined as sporulation for the purposes of the manuscript and this review) of the important pathogen Bacillus anthracis. Prior to this study, little was known about the genes required for sporulation in this pathogen or the related Bacillus cereus group because it had long been assumed that the sporulation program would be essentially the same in B. anthracis as in B. subtilis. However, as the authors point out, B. anthracis and B. subtilis diverged over 800 million years ago, and consistent with this phylogenetic divergence, they find that one-third of the genes important for sporulation in B. anthracis are not important in B. subtilis and that at least ~15% of the genes that modulate sporulation in B. anthracis are not conserved in B. subtilis. After constructing an ordered TnSeq library for B. anthracis, a notable feat in itself, the authors analyze the roles of several widely conserved sporulation genes, as well as new sporulation genes identified in their screen. Impressively, they delineate the mechanism of one of these novel sporulation genes by demonstrating that the gene encoding IpdA, which they name for "Inhibitor of Polysaccharide Deacetylases," directly binds to the polysaccharide deacetylase PdaN and likely antagonizes the activity of this peptidoglycan-modifying enzyme. Their data suggest that preventing the deacetylation of polar septum peptidoglycan promotes the activity of the peptidoglycan hydrolases responsible for driving engulfment. Excitingly, IpdA homologs are found in a range of organisms, with their phylogenetic analyses suggesting that the IpdA scaffold may generally inhibit enzymes that modify peptidoglycan. Collectively, their detailed mechanistic study of IpdA and comparative analysis of sporulation in B. anthracis and B. subtilis represent a major contribution to the sporulation field.

The manuscript is well-written, and the work presented has been conducted rigorously. I particularly appreciated the Discussion section, which is thought-provoking and effectively highlights the broader implications of their findings, such as the broader role of IseA homologs and how their comparative analyses may identify "hard-wired" vs. more "plastic" processes during sporulation. Finally, they propose that differences in cell envelope composition drives some of the organism-specific adaptations identified in their study.

We thank the reviewer for their enthusiastic response to our manuscript. We have modified the text as suggested by the reviewer (described below). In addition, we have included a few additional experiments that confirm and extend our analysis of IpdA. We include a synopsis of these experiments and the figures at the end of this file.

I have only minor comments related to the presentation and discussion of the authors' results.

-For the discussion of the results in Figure 3, I would suggest breaking up the paragraph into discrete paragraphs (line 331 after "spore"; line 336 after "discovered," and line 341 after "nutrients." It was a bit hard to keep track of all the phenotypes as written.
We have split up this long paragraph as suggested. (thank you)

- I would also suggest reordering the microscopy images to match the order in which they are described in the text. Specifically, the yhfN mutant should move before the ysxE strain.
We have re-ordered the images.

- Providing different-colored carets for the various phenotypes highlighted (e.g., circular spores vs. distorted forespores) would be helpful.
We have added different color carets as suggested.

- The authors should provide scale bars on the images where they are zoomed in. Adding a small gap between the zoomed-in region of the dual reporter fluorescence microscopy image (between the third and fourth columns) would allow the reader to more quickly recognize that the 5.75 hr phase-contrast images are distinct from the fluorescence microscopy images shown to the left.
Done!

- Do the authors know what the dark spots observed by phase-contrast microscopy in Figure 3 represent/ reflect?
We were also struck by these. We think they are storage granules. We have not (yet) pursued this.

-Is the yyaC mutant more prone to lysis? It looks like some vegetative cells may already be lysing? Did the authors see a decrease in vegetative cell counts for any of the mutants analyzed?
The reviewer has good eyes. This mutant does have more cell lysis at late stages of sporulation than others. It was the only mutant that had this phenotype. We now mention this phenotype in the text. Of note, it did not significantly reduce vegetative cell counts.

-Did the transposon used by the author have an outward-facing promoter? Would they expect their transposon insertions to have a polar effect on the expression of downstream genes? Please provide some of this information in the Methods and/or Results section.
The transposon does not have an outward facing promoter. However, the promoter of the *spec(R)* cassette is strong enough (and the terminator weak enough) that, in most cases, it provides sufficient transcription of downstream genes - provided it is oriented properly. We know this empirically because Tn insertions in operons that have downstream essential genes are tolerated in one orientation but not the other. We have added this information to the methods.

-Can the authors speculate on why the B. anthracis spoIIE mutant forms multiple septa relative to a B. subtilis spoIIE mutant.
We wonder if there is greater division potential in *B. anthracis* sporulating cells compared to *B. subtilis* - perhaps due to the expression of *ftsAZ* under Spo0A control. However, based on the comments of Reviewer 3, we have toned down our description of this difference.

- For the membrane bulge observed in engulfment mutants, the authors should consider referencing the finding by Lopez-Garrido et al. (2018), which indicates that forespore shape is influenced by the translocation of the chromosome into the forespore.
This is a terrific paper that provides a mechanism for the septal bulge phenotype and we thank the reviewer for suggesting it. However, we have decided not to include it because the level of mechanistic detail weighs down an already dense text.

- Is the membrane bulging in the B. anthracis spoIID mutant less frequent than some of the other mutants identified?
We were surprised by the lack of membrane bulging in the *B. anthracis* spoIID::Tn mutant. This is such a classic phenotype in *B. subtilis*. Other than the *ipdA::Tn* (and Δ*ipdA*) we did not observe membrane bulging in other mutants and we did not analyze any other known engulfment mutants. (see below)

- Does a B. anthracis spoIIP mutant generate membrane bulges similar to the ipdA mutant (or are the bulges less frequent like in a spoIID mutant?)
B. anthracis has two SpoIIP paralogs. Accordingly, neither came up as hits in our sporulation Tn-seq screen. We did not generate the double mutant to investigate this phenotype.

- Do the authors know whether PdaN or IpdA specifically localize to the polar septum? I am wondering if deacetylation is restricted to this site or whether all vegetative peptidoglycan is no longer deacetylated upon IpdA production.
We do not. We think this is an interesting question and merits further investigation in the future.

Line 143: please hyphenate "sporulation-defective".
Done.

Line 269: consider adding a semi-colon after "engulfment" so that it reads "… engulfment,; however, unlike…"
Done.

Line 319: the phrasing "we liked the idea that…" might be a bit colloquial for a paper.
We humbly request keeping this somewhat colloquial phrase. It is an accurate statement and think it will resonate with readers.

Line 332: "heterogeneous" has been misspelled.
Corrected (thank you).

Line 382: I think "investigated" should be "investigate"
Corrected.

Line 411: Please remove the "s" after "models".
Modified.

Line 543: "anthracis" is misspelled
Corrected.

Figure 4 legend: "false" is misspelled
Corrected.

Reviewer #2:
In this study Ramirez-Guadiana et al. determined and analyzed the molecular basis of sporulation in Bacillus anthracis and compare these findings to what is known for the sporulation model organism Bacillus subtilis. The authors identified 159 putative sporulation genes by Tn-insertion analysis using NGS. Deeper analysis revealed 58 putative sporulation genes present in B. anthracis but not previously implicated in sporulation in B. subtilis. As an example, the authors then characterized one candidate B. anthracis protein termed IpdA encoded by the gene BA_04167 in detail. Thus, this study keeps the door open to similar explorations of functions of candidate genes/proteins left unattended herein.

General comments:
1) In my opinion, this study is an excellent contribution to our understanding of which genes/proteins are surely/likely/possibly involved in sporulation of the notorious biothreat agent B. anthracis. The strength of the approach described here is the combination of global methods such as transposon-mutagenesis and omics (NGS of Tn-mutants) as well as genetic manipulation (directed mutant analysis) followed by careful observation by fluorescence microscopy for phenotypic analysis and in silico structural biology approaches.
2) At the same time, the manuscript remains easy to read (though it puts a significant - positive - strain on the reader's stamina for its volume of text and data).
3) Overall, I encountered only minor issues related to writing or data presentation as outlined

in detail below.

We thank this reviewer for their positive review of our manuscript. As mentioned above, in the revised manuscript, we include additional experiments focused on IpdA that confirm and extend our original conclusions. These are summarized at the end of the point-by-point response.

Specific (minor) comments:

4) Line 34: "new sporulation genes" is too vague. Better: "newly identified…in B. anthracis".
Changed.

5) L 37: better "…deacetylase PdaN in B. anthracis".
We think "B. anthracis" is not required here.

6) L 45: better "B. anthracis endospores are the infectious biological entities that cause the zoonotic disease anthrax".
Changed.

7) L 51-53: better "Environmental fluctuations possibly due to global climate change, or human activities such as deforestation…to the discovery of new…".
We modified this sentence to include these points.

8) L 54-56: this section is very vague and concludes with a hollow statement not matching the quality of the rest of the manuscript text. I suggest the authors formulating a scientific rationale for studying sporulation in the high-consequence spore former B. anthracis. Compare L 118-121 for a well formulated statement.
We don't find this sentence hollow and think it strikes the appropriate tone.

9) L 78: "it" is vague, please use a specific sentence object such as "the mature spore" instead.
Modified.

10) L 109 (and elsewhere): why only "~70% of the non-essential genes in B. anthracis"?
We screened ~52,000 independent transposants and only identified insertions in 70% of the non-essential genes. Our data suggest this is due to (1) limited insertions in small genes; (2) insufficient address information in the pools to allow mapping; (3) insertion hotspots; and (4) insertional bias in the origin-proximal half of the genome. We mention these in the Methods.

11) L 110: better: "…Tn mutations[sic!] that, in B. subtilis, are known to impair polar division…". Please check correct use of "mutant" and "mutation" throughout the text (for instance see L 220).
Corrected.

12) L 120: better "…and likely enable deeper…".
We think qualifying "enable" is unnecessary.

13) L 139: since "PA" has not been introduced at this point, better "…phage assay medium…".
Added (thank you).

14) L 151 (and elsewhere): does "at the onset of sporulation" essentially mean: before cells have initiated sporulation?

Thank you for noticing this. We have changed this to the onset of starvation.

15) L 159 (and elsewhere): "(≤5% of wild-type)" does certainly always mean "(≤5% sporulation efficiency compared to wild-type) [L 176]", right? Then please complete the term where needed.
Completed.

16) (L 177: there is a "the" missing before "cut off".)
Added

17) L 222-225: what about the other 9 (i.e., 62 out of 71) hits?
The remaining 9 mutants had sporulation efficiencies higher than 40% compared to wild-type and are listed in the Tables S2-S6 as indicated in the text. (It is not uncommon for a subset of hits in a Tn-seq screen to have more modest phenotypes than expected.)

18) (L 344: "…had strong sporulation defects. These mutants failed to mature…".)
Changed.

19) L 370-375: for Fig. 4A-C the order of their first reference in the text is out of order.
We have rearranged the panels in Fig. 4 to maintain the proper order.

20) (L 411: "An average model…")
Corrected

21) L 452, 454 and 456: meant here instead: "mutant protein" or "variant protein".
Thank you. Corrected.

22) (L 514: "…thought to be sensed…".
Corrected.

23) Fig. 5I is not mentioned in the text.
Citation added.

24) (L 592: "…derivative of B. anthracis…")
Corrected

25) References: make sure to format the text (e.g., italicize species names).
Formatted per PLoS Biology requirements.

26) Figure 2: add to caption the information that close up figures are close ups of the figures right above, respectively.
Added.

27) Figure 3: the percentages of sporulation given do not seem to match the visual observation of the images for ysxE::Tn and ytxC::Tn.
Both the microscopy and heat-resistance assay were performed on three biological replicates and we got similar results each time. We suspect that the spores in the images are not fully mature, heat-sensitive, or impaired in germination or outgrowth as discussed in the Results.

The last two lines of the figure legend are descriptive results and should be restricted to the results text section.
Modified.

Out of curiosity: the ysxE::Tn and ytxC::Tn mutants have low sporulation efficiencies but there are some spores. Have you repeatedly cultivated these mutants over several sporulation cycles to see if secondary mutations compensate for the Tn-induced defect?
We have not yet performed this experiment, but it is on our list.

28) Figure 4: in the figure legend, row 1 and 2 of panel A should be better explained. Lines 8 and 14-15, respectively, of the figure legend are descriptive results and should be restricted to the results text section.
We have added captions in the panel A and have modified the legend as requested.

29) Figure 5: lines 11-12 and 13-14, respectively, of the figure legend are descriptive results and should be restricted to the results text section.
Modified.

30) Legends of supplementary figures: There are descriptive results here as well.
We have removed most of the descriptions. We kept a few descriptions in the supplemental figures legends to make the figures more clear.

31) Figure S6B is missing a scale bar.
Added.

Reviewer #3: [identifies himself as Patrick Eichenberger]
This article by Ramirez-Guadiana et al reports a Tn-seq screen aimed at identifying genes required for efficient sporulation in the bacterial pathogen Bacillus anthracis. It is a follow-up to a paper by the same group (Meeske et al 2016 PLOS Biol) that applied a similar technique to the study of spore formation in the model organism Bacillus subtilis. The study is impressive by its scope and thoroughness. One of the main conclusions is that there is a significant overlap between genes required for sporulation in both organisms, thus justifying the use of model organisms that are more amenable to genetic manipulation for the study of complex developmental processes. Nevertheless, the study also demonstrated that there are several species-specific differences that merit investigation directly in the pathogen. As proof-of-concept, the last section of the article focuses on the characterization of ipdA, a gene of previously unknown function that is necessary for sporulation in B. anthracis but absent in B. subtilis. In general, the experiments are very well done with reasonable interpretation of the data. The necessary controls were included. I have a few suggestions listed below, but my comments are mostly minor.

We thank the reviewer for their positive response to our manuscript. We have modified the text as suggested (described below). In addition, we have included a few additional experiments related to IpdA and the impact of deacetylation on engulfment. We include a synopsis of these experiments and the figures at the end of this file.

Main point:
I think it is important to define exactly what is meant by the term "sporulation gene" in the context of the paper as there are many potential definitions. The authors state that there are 159 putative sporulation genes in B. anthracis. However, this is based primarily on heat resistance, which is only one phenotypic category. I would argue that the number is in fact considerably higher than this, based on transcriptomics. A "sporulation gene" could also reasonably be defined as a gene expressed during sporulation, whose expression is dependent on a sporulation transcription factor. Furthermore, a large functional category of sporulation genes, those that encode coat and exosporium proteins, is left out of the total when heat resistance is

the main criterion, because the spore coat is dispensable for heat resistance, but these genes are key for other functions. In fact, it is already known from previous work that the composition and morphology of the spore coat is one of the
main differences between all species of endospore formers. However, it would not be easy to identify coat genes in a Tn-seq approach, because there is no simple way to screen for them as some of the phenotypes are subtle. Nevertheless, I think that it would be helpful to comment on that limitation in the paper.

We thank the reviewer for asking us to elaborate. We have clarified in the text that we use the term sporulation gene to define genes that, when mutated, are impaired in spore formation as assayed by heat resistance, germination, and/or outgrowth. In the Discussion we added half a paragraph highlighting the limitations of our approach. We specifically mention genes encoding coat and exosporium proteins would not have been identified in our Tn-seq screen. We think the Discussion is improved by including this point.

Minor points:
l.81 "Although the mother" Should be "mother cell", not just "mother"
Corrected.

l.193 "Our Tn-seq screen identified 58 putative sporulation genes in B. anthracis that are present in B. subtilis but have not been reported to be required for efficient sporulation in the model (yellow circles in Fig. 1C and Table S5)." This is an oversimplification. While it is true that these genes were not identified in the B. subtilis Tn seq screen (2016 paper mentioned above), some of these genes had already been identified as genes required for sporulation in B. subtilis. For example, ypjB, yhbH and prkA are all featured as SigE-dependent genes that are necessary for heat-kill resistance in B. subtilis in Ref 16 (albeit with modest phenotypes). So, the statement that "25 of them are induced during B. subtilis sporulation but lack reportable phenotypes [16, 29]" is not a fully accurate representation
of the previous work.

We have modified the text to make this point. We thank the reviewer for pushing us to be more accurate.

l. 236 "transcriptional reports" should be "transcriptional reporters"
Corrected.

l.251 "These pleiotropic phenotypes have not been reported for the B. subtilis spoIIE mutant and hint at differences between the two species." I would be more cautious here. The fact that they have not been reported does not mean that they don't exist in B. subtilis (in fact, I know from personal experience that a similar phenotype can be observed in a spoIIE mutant in B. subtilis after a few hours). It is fine to report the phenotype, but I would not infer that it signals major differences between the two species.
We thank the reviewer for mentioning their unpublished results. We have toned down this section.

l.270 "mother membranes" should be "mother cell membranes".
Changed.

l.345 "ysxE" You could mention here that YsxE has been characterized as an inner spore coat protein in B. subtilis (McKenney et al. 2010, Current Biology). Furthermore, since ysxE is part of the spoVID operon, it may play a role in a SpoVID checkpoint in B. anthracis similar to the

mechanism described in B. subtilis by Delerue et al. (2022, Dev Cell).
Not sure that panel A in Fig.1 is necessary (could be moved to the suppl)
YsxE was shown as an example of a mutant with a strong sporulation defect that lacked a morphological phenotype. Since we don't discuss this mutant beyond making this point, we have decided not to include this information.

Brief synopsis of additional results that are included in the resubmission:

Background:
In our original submission, we identified loss-of-function mutations in *pdaN* and *pcdF* as suppressors of a deletion in the sporulation factor IpdA (**Fig. 5B and 5C**). The *pdaN* gene encodes the polysaccharide deacetylase PdaN. AlphaFold predicts that IpdA binds and inhibits the catalytic pocket of PdaN (**Fig. 5D**). It also predicts that PdaN and PdcF interact (**Fig 5E**). Based on the predicted interaction between PdaN and PdcF and that mutations in either gene suppresses Δ*ipdA*, we hypothesized that PdaN and PdcF function together (**Fig. 5F**).

New Results:
In the revised manuscript we show that co-expression of *B. anthracis pdaN* and *pdcF* in *B. subtilis* impairs growth (**Fig. S16A**). Importantly, this growth defect requires expression of both *pdaN* and *pdcF*, providing further evidence that the encoded proteins function together. Furthermore, we visualized the *B. subtilis* cells induced to express PdaN and PdcF and discovered that the cells become small, chubby, and curved, and ultimately lyse (**Fig. 5G and S16B**). These morphological phenotypes are strikingly similar to the phenotypes associated with depletion of the peptidoglycan hydrolases (CwlO and LytE) that are required for cell wall elongation. The papers we reference (*Meisner et al. 2013*, *Brunnet et al. 2019*) come from our lab and the morphological defects are practically identical to the ones we report here. In a third publication (*Dobihal et al. 2022*), we showed that overexpression of a *B. subtilis* peptidoglycan deacetylase PdaC impairs CwlO and LytE cleavage of the peptidoglycan. Collectively, these data argue that *B. anthracis* PdaN/PdcF work together to deacetylate cell wall peptidoglycan and this modification prevents cleavage by cell wall remodeling enzymes. Finally, we investigated whether expression of PdaN and PdcF in *B. subtilis* at the onset of sporulation impairs engulfment in a manner similar to the Δ*ipdA* mutant in *B. anthracis*. We found that the phenotypes are almost indistinguishable (**Fig. 5K and S18**). The *B. subtilis* septal membrane bulges into the mother cell cytoplasm provided both *B. anthracis* PdaN and PdcF are expressed. These data support our model that the SpoIID and SpoIIP cell wall hydrolases that mediate the phagocytic process of engulfment are unable to cleave deacetylated peptidoglycan.

Finally, we noted in the original submission that a *pdcF::Tn* mutant suppresses the engulfment defect of the Δ*ipdA* deletion better than the *pdaN::Tn* mutant (**Fig. 5H**). We hypothesized that PdcF functions as a co-factor for additional polysaccharide deacetylases. In this model, sporulating cells lacking PdcF (*pdcF::Tn*) have fewer active deacetylases than *pdaN::Tn*, resulting in more acetylated septal peptidoglycan, more cleavage/remodeling by the engulfment hydrolases, and more suppression. To explore this model further, we investigated whether the *B. anthracis pdcF::Tn* mutant was more sensitive to lysozyme. *B. anthracis* is highly resistant to lysozyme. It can grow in concentrations of 1 mg/mL. For comparison, *B. subtilis* is unable to grow in the presence of 2 µg/mL (500-fold less). Work from the 1980s using purified *B. anthracis* cell wall sacculi showed that lysozyme resistance was due to the extensive deacetylation of GlcNAc and MurNAc sugars in the *B. anthracis* peptidoglycan. We found that the *pdcF::Tn* mutant was more sensitive to lysozyme than *pdaN::Tn* or wild-type. (**Fig. S17**). This result supports our model that PdcF is a co-factor for several deacetylases, and in its absence, the glycan strands are more acetylated resulting in greater susceptibity to lysozyme.

Collectively, these new results provide additional support for our model that IpdA induction at the onset of sporulation inhibits the PdaN/PdcF deacetylase complex as well as other deacetylase/PdcF complexes. This inhibition ensures that the nascent peptidoglycan at the polar septum and likely the proximal layer(s) that surround the spore are acetylated and therefore can be acted upon by the engulfment hydrolases (SpoIID and SpoIIP) to promote proper engulfment of the developing spore. With these new results, we decided a model figure was appropriate and added it to the main text (**Fig. 6A**).

We have attached the figures referenced above with all new figures boxed in red.

-David and Fer

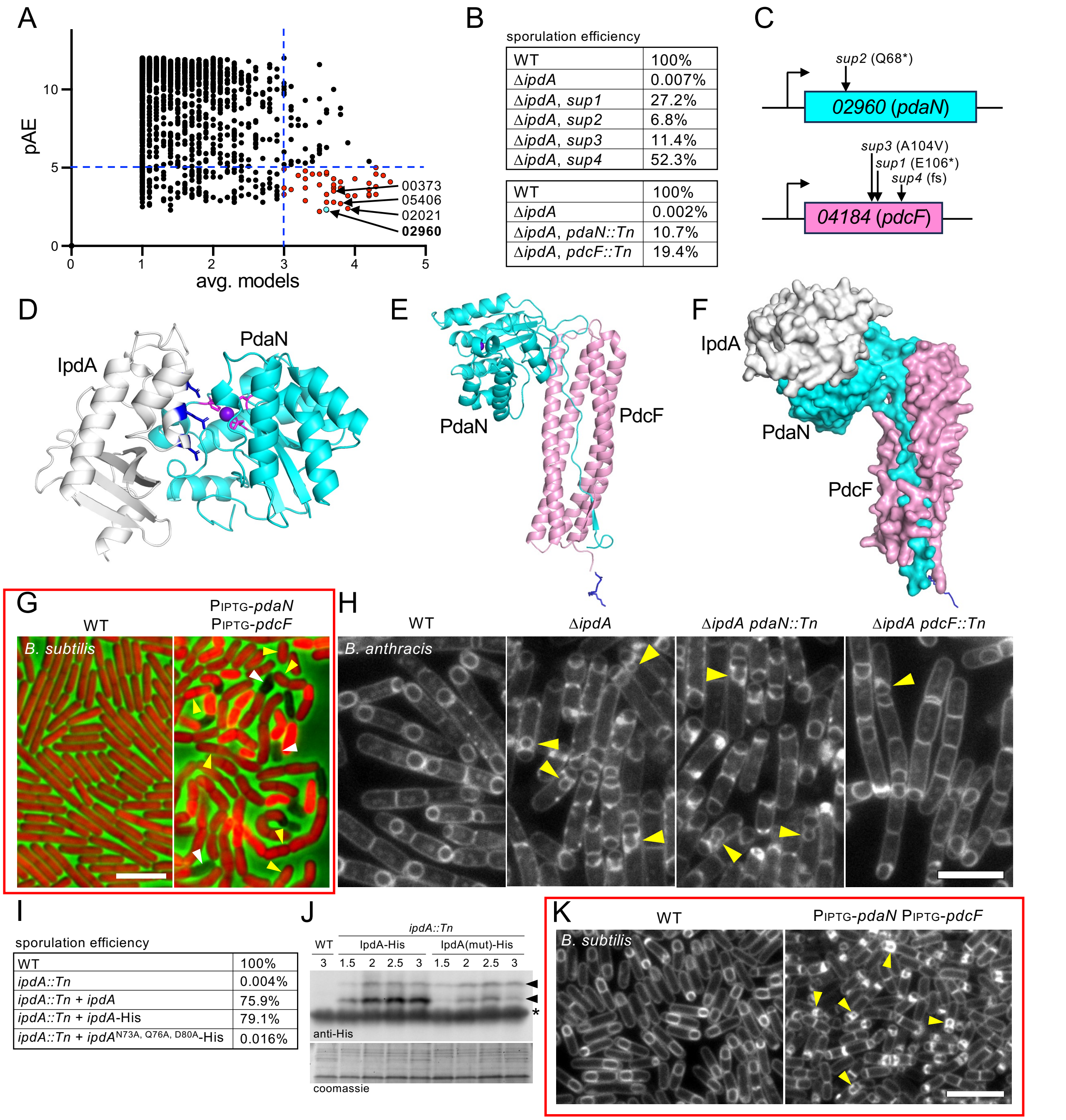

Figure 5. IpdA inhibits the polysaccharide deacetylase PdaN (02960). (A) Scatterplot of all potential hits (circles) from a one-by-all AlphaFold2-Multimer screen using IpdA as the bait. Five models were predicted for each protein in the *B. anthracis* proteome. All proteins with an average model score ≥1 are plotted against the average pAE score from the best model. Dashed blue lines show arbitrarily assigned significance thresholds (pAE ≤5, average models ≥3). 44 proteins met these criteria. 02960 (PdaN) is shown in cyan. Three other polysaccharide deacetylases are also indicated. **(B)** Sporulation efficiency of suppressors of Δ*ipdA* (top) identified through an enrichment screen (see Methods). Validation of the hits from the screen using transposon-insertion mutants (bottom). **(C)** Schematics of the *02960* (*pdaN*) and *04184* (*pdcF*) genes with suppressor mutations highlighted. **(D)** AlphaFold3-predicted complex of IpdA (white) and the catalytic domain of PdaN (cyan). PdaN's catalytic residues (magenta) and Zn^{2+} ligand (purple sphere) are shown. Residues in IpdA predicted to interact with PdaN are shown in dark blue. **(E)** AlphaFold3-predicted complex of PdaN (cyan) and PdcF (pink). The predicted lipidated N-terminus is shown in dark blue. **(F)** AlphaFold3-predicted complex of IpdA (white), PdaN (cyan), PdcF (pink). The pAE plots for Figs. 5D-F can be found in Figures S12 and S13. **(G)** Representative fluorescence images of the indicated *B. subtilis* strains with cytoplasmic mCherry (red) 3 h after addition of IPTG (500 µM, final) to induce expression of *B. anthracis pdaN* and *pdcF*. Short, chubby cells (yellow carets) that ultimately lyse (white carets) are highlighted. A complete time course can be found in Figure S16. **(H)** Representative fluorescence images of the indicated *B. anthracis* strains at hour 4 of sporulation. Yellow carets highlight sporulating cells with septal bulges. *pdaN::Tn* modestly suppresses the morphological defects associated with Δ*ipdA* despite the 500-fold suppression of the sporulation defect. *pdcF::Tn* suppresses the septal bulging to a greater extent. **(I)** Sporulation efficiencies of the indicated strains. An IpdA variant with three amino acid substitutions in residues predicted to interact with PdaN fails to complement the Δ*ipdA* mutant. **(J)** Representative anti-His immunoblot of the indicated strains over a sporulation time course. Time in hours after the onset of sporulation is indicated above the blots. Black carets highlight the bands specific to IpdA-His. The lower band (highlighted with an asterisk) is lysozyme, used to generate the lysates. A coomassie-stained gel was used to control for loading. **(K)** Representative fluorescence images of the indicated *B. subtilis* strains at hour 4 of sporulation. IPTG (500 µM, final) was added to the cultures at the onset of sporulation. Yellow carets highlight septal bulges. A complete time course can be found in Figure S18. Scale bars indicate 5 µm. The data underlying panels A, B and I can be found in S1 Data.

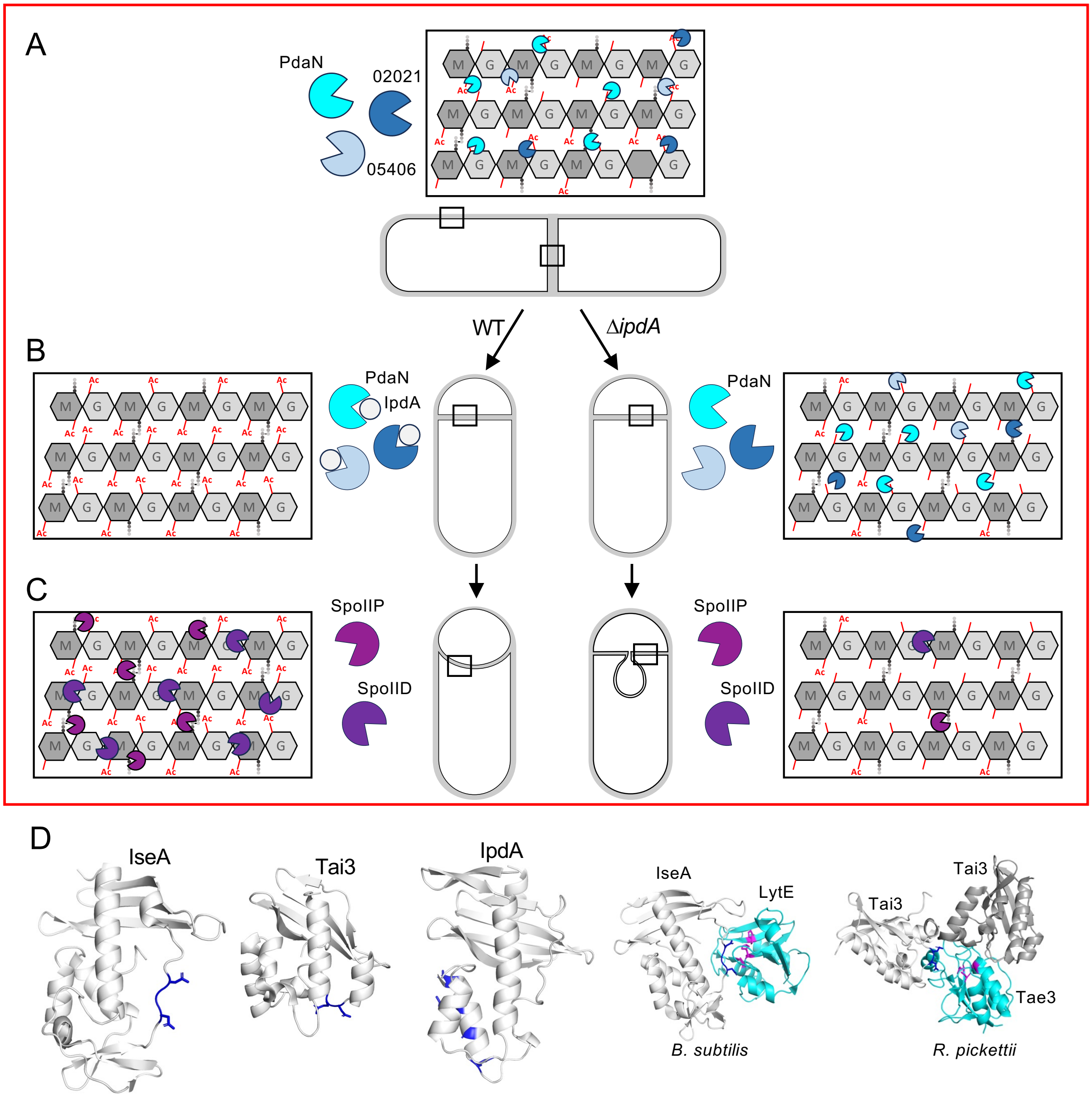

Figure 6. Schematic model for IpdA function during sporulation in *B. anthracis*. (A) During vegetative growth, PdaN, 02021, 05406 are proposed to deacetylate the N-linked acetyl groups from GlcNAc (G) and MurNAc (M) sugars of the peptidoglycan (PG). **(B)** At the onset of sporulation but prior to polar division, IpdA is induced and inhibits deacetylase activity, resulting in acetylated septal PG (left). In the Δ*ipdA* mutant, the septal PG is deacetylated (right). **(C)** The cell wall hydrolases SpoIID and SpoIIP can cleave acetylated PG and promote engulfment (left). SpoIID and SpoIIP are unable to efficiently cleave deacetylated PG in the Δ*ipdA* mutant, resulting in septal membrane bulges (right). For simplicity, the deacetylase co-factor PcdF is not shown. **(D)** Comparison of IseA homologs from *B. subtilis*, *Ralstonia pickettii* and *B. anthracis.* The region on each protein that interacts with its cognate enzyme active site is colored dark blue. Structures of IseA-LytE (PDB: 8I2E) and Tai3:Tae3 (PDB: 4HZ9) are shown on the right. Only the catalytic domains of LytE and Tae3 are depicted.

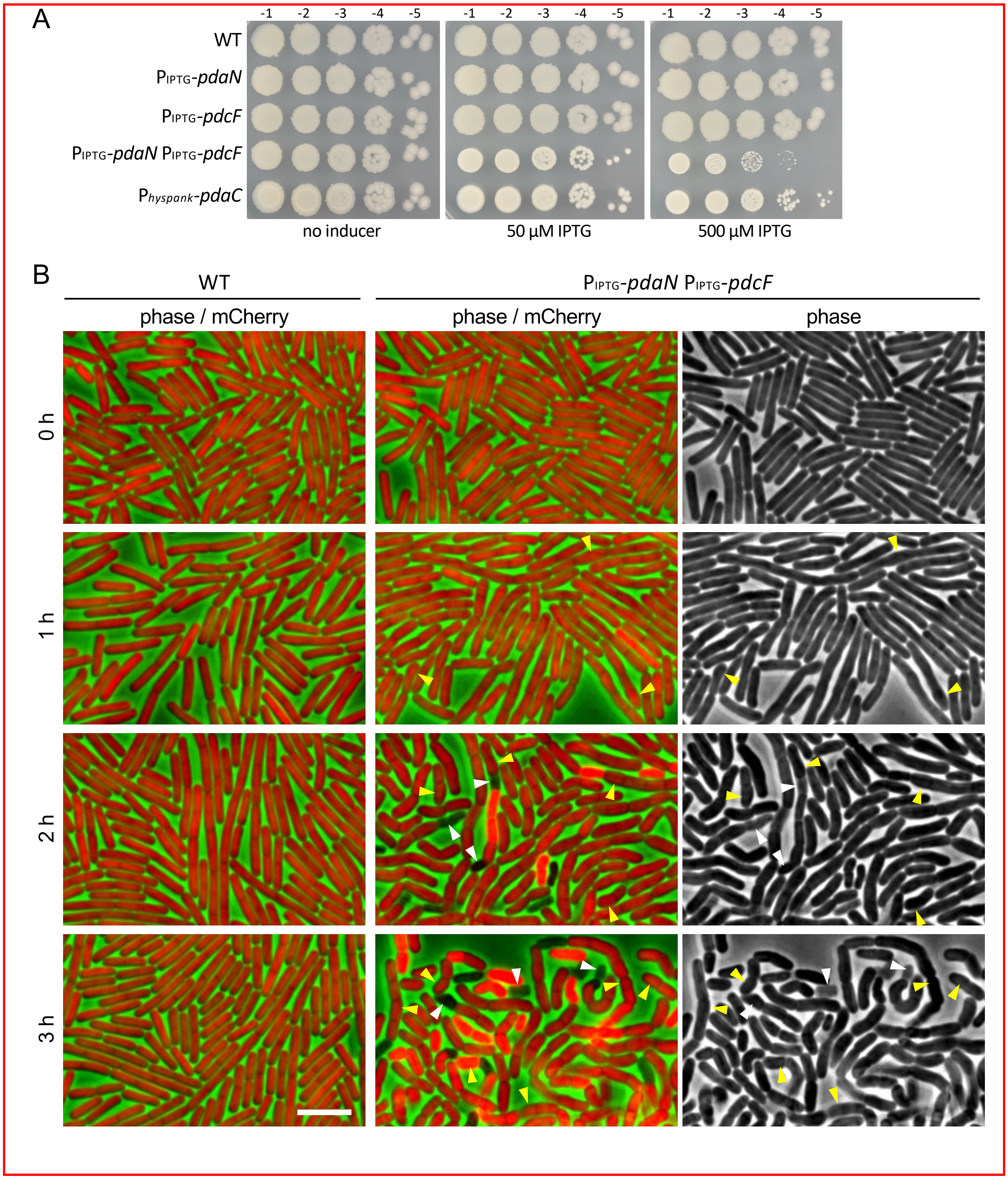

S16 Fig. Expression of *B. anthracis pdaN* and *pdcF* in *B. subtilis* inhibits cell elongation. (A) Photographs of spot-dilutions of the indicated *B. subtilis* strains on LB agar plates. 10-fold serial dilutions of the indicated strains were spotted on LB agar supplemented without or with 50 µM or 500 µM IPTG. The *pdaN* and *pdcF* genes were fused to the P_{spank} (P_{IPTG}) promoter. The *B. subtilis pdaC* gene was fused to the $P_{hyperspank}$ ($P_{hyspank}$) promoter. The $P_{hyperspank}$ promoter is ~7-fold stronger than the P_{spank} promoter. **(B)** Representative fluorescence and phase-contrast images of exponentially growing cells before and after addition of IPTG. Exponentially growing cultures of the indicated strains expressing cytoplasmic mCherry (red) were analyzed at the indicated times after IPTG (500 µM, final) addition. Short, chubby cells (yellow carets) and lysed cells (white carets) are characteristic of inhibition of cell wall elongation. Scale bar indicates 5 µm.

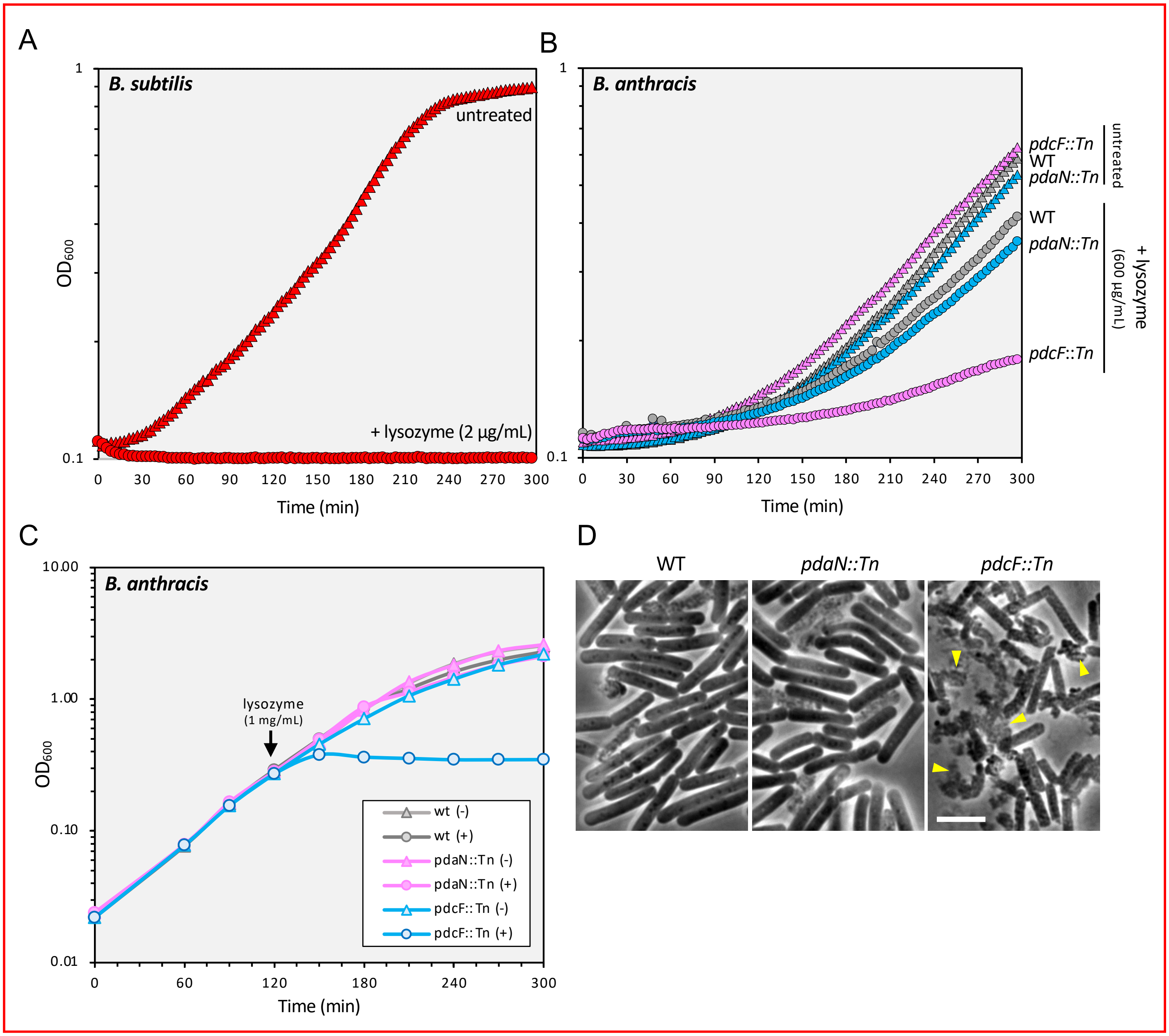

S17 Fig. *B. anthracis* cells lacking *pdcF* are more sensitive to lysozyme. Growth curves of *B. subtilis* (**A**) and *B. anthracis* (**B**) in the presence of lysozyme (2 µg/mL for *B. subtilis;* 600 µg/mL for *B. anthracis*). The indicated strains were grown in sporulation media until mid-exponential phase and back-diluted into fresh media with and without the indicated concentrations of lysozyme. OD_{600} was recorded every 3 min for 5 h. **(C)** Growth curve of the indicated *B. anthracis* strains before and after acute exposure to 1 mg/mL lysozyme (arrow). The indicated strains were grown in sporulation media until mid-exponential phase and back-diluted to an OD_{600} of 0.05. When the cultures reached an OD_{600} ~0.3, they were divided in two. One subculture was exposed to lysozyme (1 mg/mL, final) and the other was left untreated. OD_{600} was recorded every 30 min for 3 h. **(D)** Representative phase-contrast images of the indicated strains in (C) 3 h after addition of lysozyme (1 mg/mL, final). Yellow carets highlight lysed cells. Scale bar indicates 5µm. The data underlying panels A, B and C can be found in S1 Data.

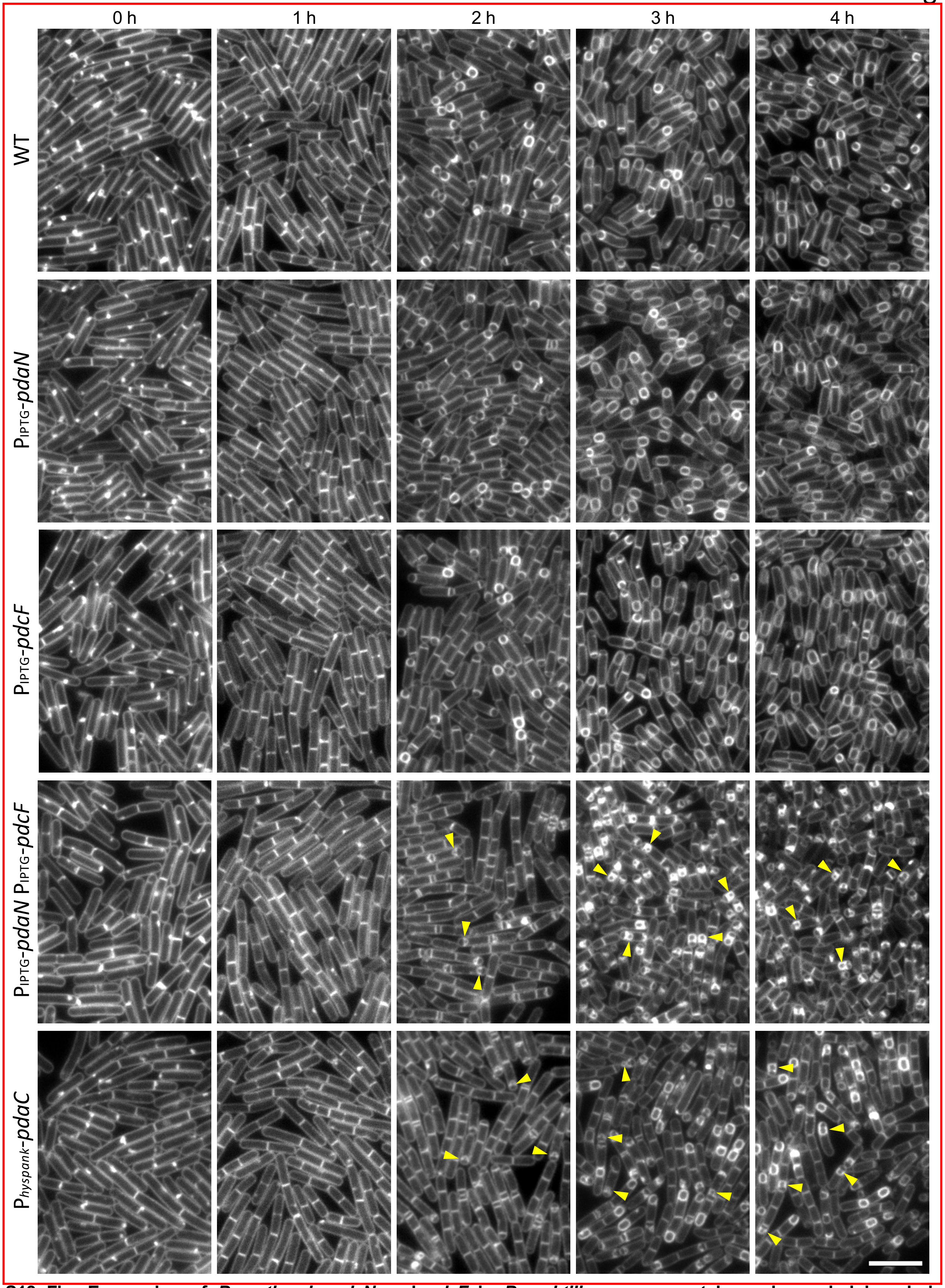

S18 Fig. Expression of *B. anthracis pdaN* and *pdcF* in *B. subtilis* causes septal membrane bulging during sporulation. Representative images of the indicated strains during a sporulation time course. Sporulation was induced by the resuspension method, and IPTG (500 µM, final) was added to the cultures at the time of resuspension (0 h). Cells were examined by fluorescence microscopy at the indicated time points. Septal bulges (yellow carets) are highlighted. Scale bar indicates 5 µm. Expression of *B. subtilis* PdaC, a membrane-anchored MurNAc deacetylase that is not normally produced during sporulation, was included for comparison.
